# Immunopathology in PMM2-CDG: Defective glycosylation impact in the TNFα -TNFR1 signalling pathway

**DOI:** 10.3389/fimmu.2025.1655354

**Published:** 2025-09-18

**Authors:** Carlota Pascoal, Pedro Granjo, Rebeka Kodríková, Marta Falcão, Ana C. Santos, Inês Teodoro, Zuzana Pakanová, Marek Nemčovič, Jan Mucha, Margarida Castro-Caldas, Ana R. Grosso, Vanessa dos Reis Ferreira, Paula A. Videira

**Affiliations:** ^1^ UCIBIO – Applied Molecular Biosciences Unit, Department of Life Sciences, NOVA School of Science and Technology, Universidade NOVA de Lisboa, Caparica, Portugal; ^2^ Associate Laboratory i4HB - Institute for Health and Bioeconomy, NOVA School of Science and Technology, Universidade NOVA de Lisboa, Caparica, Portugal; ^3^ CDG and Allies-Professionals and Patient Associations International Network, Caparica, Portugal; ^4^ Institute of Chemistry, Slovak Academy of Sciences, Bratislava, Slovakia; ^5^ Research Institute for Medicines (iMed), Faculty of Pharmacy, Universidade de Lisboa, Lisboa, Portugal

**Keywords:** TNF-α, fibroblasts, PMM2-CDG, inflammation, transcriptomics, glycomics

## Abstract

**Introduction:**

Glycosylation is a post-translational modification that plays a crucial role in immune system activity. Phosphomannomutase 2-Congenital Disorder of Glycosylation (PMM2-CDG) is a rare genetic disease affecting glycosylation with a multi-systemic impact. PMM2-CDG patients commonly show immune disfunction and elevated pro-inflammatory cytokine levels that may link to other symptoms. However, the underlying immune mechanisms remain unclear. Given Tumour Necrosis Factor (TNF)’s key role in inflammation, this study proposes that defective glycosylation of its receptors disrupts intracellular signalling, leading to changes in the immune response of PMM2-CDG patients.

**Methods:**

To address this, we applied an integrative approach, combining transcriptomics, glycomics, and immune-related assays to investigate the impact of TNF-a stimulation via TNF receptor 1 (TNFR1) in a cohort of PMM2-CDG patients’ skin fibroblasts.

**Results:**

Our results reveal a multifaceted disruption of TNF-a signalling in PMM2-CDG fibroblasts. We observed structural abnormalities in TNFR1, including altered receptor shedding. PMM2-CDG cells also showed an altered N-glycosylation profile, affecting particularly, high mannose N-glycans. At transcriptional level, PMM2-CDG cells, especially those bearing the R141H heterozygous variant, exhibited a distinct gene expression profile, after stimulation, characterized by dysregulation of immune and signalling pathways. Functionally, these molecular alterations translated into a diminished secretion of key inflammation and infection mediators, such as interleukin-6 (IL-6) and C-C Motif chemokine ligand 5 (CCL5) upon TNF-a stimulation. Similarly, essential signalling kinases including extracellular-signal-regulated kinase (ERK) 1/2, p38 and c-Jun N- terminal kinase (JNK) 2 showed reduced expression in PMM2-CDG cells, and their expression did not alter following TNF-a stimulation, unlike control cells.

**Conslusion:**

Our findings point to TNFR1 signalling dysregulation as a key contributor to immune dysfunction in PMM2-CDG. Importantly, our study identifies TNFR1 as a promising therapeutic target, suggesting that strategies aimed at modulating TNFR1 activity or restoring glycosylation homeostasis could provide new approaches for treatment development. This work advances our understanding of PMM2 -CDG immunopathology and opens opportunities for targeted therapeutics.

## Graphical Abstract

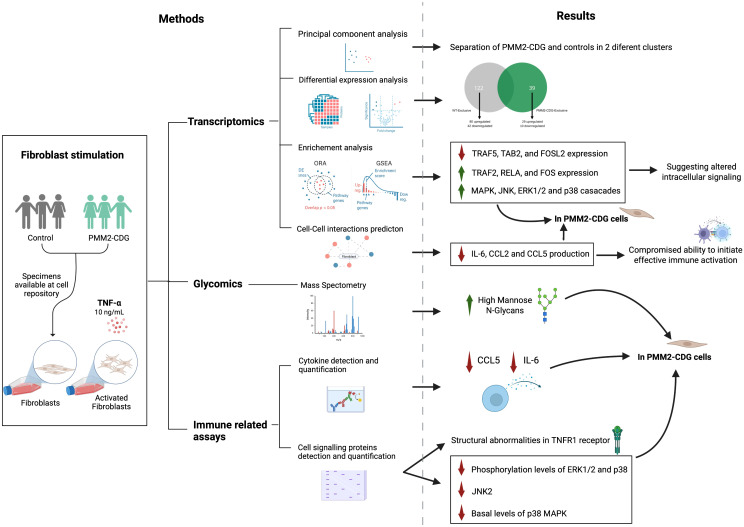

## 1 Introduction

The immune system is a sophisticated defence network that protects the body against pathogens through a coordinated interplay of innate and adaptive responses (1). Central to these processes are glycans-complex oligosaccharide structures that decorate nearly all immune receptors and effector molecules, influencing immune recognition, activation and intercellular communication (2, 3).

Glycans exert vital structural and regulatory roles within the immune system (4), as evidenced by their involvement in pathological conditions, such as infection and autoimmune diseases (5). Congenital disorders of glycosylation (CDG), comprising approximately 190 genetic diseases caused by errors in the genes involved in glycan biosynthesis (6). These disorders often present multi-systemic manifestations (7), and while neurological manifestations are very prevalent, several CDG have been associated with immune dysfunction and even classified as immunodeficiencies (8–10).

The most frequent CDG, with more than 900 reported patients, is phosphomannomutase 2 (PMM2)-CDG (11). It results from mutations on PMM2 gene which encodes an enzyme critical for the synthesis of N-glycans (12, 13). Individuals with PMM2-CDG exhibit a wide range of clinical features and experience recurrent and severe infections that can cause hospitalization and fatalities (14). Infections or inflammatory episodes often exacerbate neurological symptoms, like seizures and stroke-like episodes significantly impacting patients’ quality of life (10, 15–19). These patients often present immunological abnormalities, including altered white blood counts, (e.g. lymphopenia, neutropenia), and hypogammaglobulinemia (17, 20–24), as well as elevated inflammatory cytokines, such as Tumour Necrosis Factor (TNF)-α and interleukins (IL) 1, 6, 8 in serum and in body fluids (25). Dysregulation in cytokine response pathways including the activation of the transcription factor, nuclear factor kappa-light-chain-enhancer of activated B cells (NF-κB), was recently acknowledged, suggesting that inflammatory signalling mechanisms contribute to disease’s pathophysiology (26, 27). Functional defects such as impaired neutrophil chemotaxis, increased natural killer (NK) cell reactivity (21) and hypoglycosylation of immune receptors have also been reported (28). However, the molecular mechanisms underlying immune dysfunction in PMM2-CDG remain poorly understood. Knowing this and giving the central role of TNF-α in coordinating inflammatory signalling covering NF-κB (29), alterations in this pathway may critically contribute to the immune abnormalities observed in PMM2-CDG patients. TNF receptor 1 (TNFR1) is a key mediator of TNF signalling. After TNF binding, TNRF1 recruit’s adaptor proteins such as TNF receptor type 1-associated death domain (TRADD), receptor-interacting serine/threonine-protein kinase 1 (RIPK1), TNF receptor associated factor 2 (TRAF2) or TRAF5, and cellular inhibitor of apoptosis protein 1 or 2 (cIAP1/2). These initiate downstream signalling cascades involving ubiquitination of RIPK1 and recruitment of transforming growth factor β (TGFβ)-activated kinase 1 (TAK1), TAK-binding protein 2 (TAB2) and TAB3, that leads to the activation of NF-κB, JUN N-terminal kinase (JNK), and p38- mitogen-activated protein kinases (MAPK) signalling pathways (30, 31). TRAF2 can also activate apoptosis signal-regulating kinase 1 (ASK1), which activates JNK and Extracellular Signal-Regulated Protein Kinase (ERK) 1/2 (32). In the absence of RIPK1 ubiquitination, apoptotic or necroptotic signalling may be activated via FAS-associated death domain (FADD), caspase 8 (CASP8), which are regulated by CASP8 and cellular FLICE inhibitory protein (cFLIP) (30).

Although these signalling pathways are well characterized, the contribution of protein glycosylation to their regulation remains poorly understood. TNFR1 contains four glycosylation sites (33), which influence stability, ligand biding, and downstream signalling efficiency (34). Despite the centrality of glycosylation in receptor function, the extent to which deficient glycosylation impairs TNFR1 mediated responses in PMM2-CDG is still unknown. To address this gap, in this study we explore the impact of deficient glycosylation in the intracellular signalling triggered by TNF-α, leading to mechanistic faults behind inflammation in PMM2-CDG. By integrating transcriptomics, glycomics and functional immune assays in patient-derived fibroblast, we aim to identify the processes and pathways most affected by defective glycosylation.

## 2 Materials and methods

Details of the reagents, equipment and software used can be found in **Supplementary Table S1**.

### 2.1 Fibroblasts acquisition, culture and stimulation

Skin fibroblasts derived from PMM2-CDG and apparently healthy individuals were acquired from the NIGMS Human Genetic Cell Repository at the Coriell Institute for Medical Research (**Table 1**). All patients were heterozygous. Cells were cultured using complete Dulbecco’s Modified Eagle Medium (1g/L glucose, 2 mM L-glutamine, penicillin-streptomycin (100 units/mL and 100µg/mL, respectively) and 10% (v/v) heat-inactivated foetal bovine serum) at 37°C in a 5% CO2 humidified incubator.

**Table 1:** Clinical data of the PMM2-CDG patients (P1 – P3) and healthy individuals (C1 – C3) included in this study.

Study ID	Coriell ID	Mutated gene	Gene variants	Gender	Age at sampling (years)	Affected Domain/Functional Consequence [revised in (35)]
P1	GM20945	PMM2	c.95TA>GC/c.470T>C (p.L32R/p.F157S)	Female	7	Folding/stability – Misfolded protein, reduced stability ^▲ ■^
P2	GM27226	PMM2	c.422G>A/c.647A>T (p.R141H/p.N216I)	Male	1	Catalytic domain – Loss of catalytic efficiency, reduced N-glycan synthesis ^▲ ■^
P3	GM27386	PMM2	c.422G>A/c.415G>A (p.R141H/p.E139K)	Female	5	Catalytic domain – Loss of catalytic efficiency, reduced N-glycan synthesis^▲^ Uncertain - High residual PMM activity and less thermolability ^■^
C1	GM00498	–	–	Male	3	
C2	GM00969	–	–	Female	2	
C3	GM03349	–	–	Male	10	

The column *Affected Domain/Functional Consequence* summarizes the predicted structural domain of the encoded protein affected by the variants (e.g., folding, dimerization, catalytic site, or loss of protein) and the expected functional impact on enzymatic activity (35). Variants are indicated as follows: ^▲^allele 1 ^■^ allele 2.

For RNA sequencing, lectin staining and protein analysis, 5 x 10^5^ fibroblasts were seeded in T75 flasks and cultured for 72 hours. Cells were then stimulated with 10 ng/ml TNF-α for either 5 hours (for RNA sequencing) or 24 hours (for lectin staining, membrane protein detection, or whole-cell lysate protein analysis). For signalling pathway and secreted protein expression assays, 2 × 10^5^ fibroblasts were seeded in 6-well plates, allowed to grow for 72h, and subsequently stimulated with 10 ng/ml TNF-α for 30 minutes or 24 hours, respectively. Following stimulation, both cell culture supernatants and cell pellets were collected, centrifuged, and either processed immediately or stored at -20 °C until further analysis.

### 2.2 Detection and quantification of PMM2, TNFR1 and signalling proteins by Western blot

Following stimulation, cells were washed with cold phosphate-buffered saline (PBS) and lysed using Pierce IP lysis buffer (ThermoFisher Scientific) supplemented with a protease inhibitor (cOmplete, Mini, EDTA-free Protease Inhibitor Cocktail, Roche), resulting in total cell lysates. For lysates intended for signalling proteins detection, Na_3_>VO_4_> was also included, according to manufacturer’s instructions. Protein concentration was determined using the Pierce BCA Protein Assay Kit (ThermoFisher Scientific), following the manufacturer’s instructions. 30 µg of total protein were separated on a 10% SDS-PAGE gel and transferred to a polyvinylidene difluoride (PVDF) membrane. Membranes were blocked with 5% non-fat dried milk and immunoblotting was carried out with antibodies against PMM2 (1:1000, 10666-1-AP, Proteintech), p38 (1:1000, sc-728, Santa Cruz Biotechnology), p-p38 (1:1000, #9211, Cell Signalling), ERK1/2 (1:1000, #9102, Cell Signalling), p-ERK1/2 (1:1000, #9101, Cell Signalling), IκBα (1:600, sc-371, Santa Cruz Biotechnology) and JNK2 (1:1000, #9258, Cell Signalling) overnight, followed by 1h incubation with the Peroxidase AffiniPure donkey anti-rabbit IgG (H+L) (1:10000, #711-035-152, Jackson Laboratories) secondary antibody.

Signal was revealed using the Lumi-Light Western Blotting Substrate (Roche) to X-ray films (Amersham HyperfilmTM ECL). For loading control, membranes were stripped using ReStore^®^ Western Blot stripping buffer (ThermoFisher Scientific) following the manufacturer’s instructions and re-probed using anti-α-tubulin antibody (1:50000, T6074, Sigma-Aldrich). Bands’ optical density was quantified using the ImageJ software (v.1.43) and normalized using the housekeeping protein normalization method (36).

For the detection of TNFR1 (H-5) (1:500, sc-8436, Santa Cruz Biotechnology), the procedure was carried out as previously describe, using HRP Goat Anti-Mouse Ig (1:2000, #554002, BD Biosciences) as the secondary antibody, except for the signal detection. In this case the chemiluminescent signal was obtained using iBright FL1500 Imaging System (ThermoFisher Scientific) with the DuoLux Chemiluminescent/Fluorescent Substrate, Peroxidase (Vector Laboratories). Membranes were re-probed with anti-α-tubulin antibody (1:10000, T6074, Sigma-Aldrich), and band intensity was quantified using the iBright Analysis Software (v 5.4.0, ThermoFisher Scientific). Data were normalized using software’s Housekeeping Protein (HKP) Normalization method with Lane Background Correction.

### 2.3 Cell phenotyping by flow cytometry

To analyse the overall cell N-glycosylation, 1x10^5^ cells were stained with Concanavalin A (ConA)-biotin (1:100, #B-1005, VectorLabs) and Galanthus nivalis Lectin (GNL)-biotin (1:100, #B-1245, VectorLabs) at 4°C for 20 min. Streptavidin-PE (1:100, #554061, BD Biosciences) was used for secondary detection. Cell surface TNFR1 staining was performed using PE anti-human CD120a antibody (1:100, #369903, Biolegend) at 4°C for 20 min. After staining, cells were washed and fixed with 2% paraformaldehyde. Data were acquired using the Attune Acoustic Focusing Cytometer (Applied Biosystems) and analysed with FlowJo software (v 10.0.5, BD Biosciences). Data were presented as delta mean fluorescent intensity (MFI) obtained by subtracting the MFI of the secondary staining control or unstained cells, as appropriate.

### 2.4 N-glycoprofiling of cell lysates by mass spectrometry

To analyse the whole N-glycoprofile, fibroblasts, stimulated and non-stimulated with TNF-α, pellets were lysed by freeze/thaw cycles, and the extracted proteins were enzymatically deglycosylated by PNGase F. Released N-glycans were isolated on Supelclean ENVI-Carb SPE columns and permethylated as previously described (37).

The permethylated N-glycans were dissolved in 50% methanol and mixed with the matrix solution of 2,5-dihydroxybenzoic acid. Samples were analysed in reflectron positive ion mode using UltrafleXtreme II MALDI-TOF mass spectrometer. All presented N-glycan structures were confirmed through MS/MS analysis (LIFT mode). Raw data were processed using FlexAnalysis v.3.4, ProteinScape v.3.0 (both from Bruker Daltonics), and GlycoWork Bench software (38).

### 2.5 Quantification of secreted cytokines using ELISA and LEGENDplex™

The concentration of IL-1β, IL-6 and IL-15 was determined by sandwich ELISA using commercial kits (**Supplementary Table S1**). Signal was quantified by measuring the absorbance at 450 nm on a SpectraMax 190 Microplate Reader. A Mix and Match LEGENDplex™ panel was used to measure C-X-C Motif Chemokine Ligand (CXCL) 1, CXCL5, CXCL8, CCL2 and CCL5. Signal was detected in the BD LSRFortessaTM X-20 Cell Analyzer (BD Biosciences). Cytokine and chemokine concentrations were obtained using the specific standard curves and normalized to the total protein concentration, quantified using the Pierce BCA Protein Assay Kit. All reagents and instruments were used following manufacturers’ instructions.

### 2.6 RNA extraction, sequencing, alignment and data availability

Fibroblasts’ total RNA was extracted using the GenElute Mammalian Total RNA Miniprep Kit (Sigma Aldrich), following the manufacturer’s instructions. NanoDrop ND-1000 Spectrophotometer (ThermoFisher Scientific) was used to evaluate the purity and concentration of the RNA samples. RNA integrity was analysed using the High Sensitivity RNA Analysis kit on Fragment Analyzer (Agilent Technologies Inc), where it was deemed acceptable if 280:260 nm and 260:230 nm ratios were higher than 1.9 and 1.5, respectively, and if the 28S:18S ratio was higher than 2.0.

cDNA libraries were prepared using the QuantSeq 3’ mRNA-Seq Library Prep Kit FWD for Illumina (Lexogen, Ghmb) by the Genomics Unit of Instituto Gulbenkian de Ciências (Oeiras, Portugal). RNA sequencing was performed on the NextSeq500 (Illumina) in a 75-base single-end mode, with a minimum target coverage of 6M reads per library. The QuantSeq 3’mRNA-Seq Integrated Data Analysis Pipeline on BlueBee^®^ Genomics Platform was employed to obtain the read counts for each sample. RNA-seq raw data quality control was assessed using the FastQC (v.0.11.5) (39) and BBDuk software (v.35.92) was used to trim and remove the standard adapter sequences and poly(A) tails (40). The reads were aligned with the reference genome (GRCh38) and counted using STAR and HTSeq, respectively (41, 42). Individual-level data is available at the database of Genotypes and Phenotypes for authorized investigators (accession number: phs003313.v1.p1).

### 2.7 Predicted cell-cell interactions between TNF-α stimulated fibroblasts and immune cells

Based on receptor-ligand pairs and ligand and receptor repertoires of 144 primary cell types from a recent article from the FANTOM 5 project (43), we examined potential interactions between fibroblast and immune cells upon TNF-α stimulus. First, we selected cell-cell interactions associated with receptors or extracellular ligands codified by the identified differentially expressed genes (DEGs) (described in the Statistical Analysis topic). Then, we assessed the gene expression of the corresponding ligand/receptor pairs in different immune cell lines, with a threshold of 10 transcripts/million (∼3 transcripts/cell), as previously described (**Supplementary Table S2**) (43, 44). The output was a list of potential interactions between our stimulated fibroblasts and several immune cells following TNF-α stimulus.

### 2.8 Statistical analysis

Statistical significance was analysed using GraphPad Prism (v.8.4.0, GraphPad LLC). Data normality was accessed by the Shapiro-Wilk test and comparisons of means between the four sample groups were analysed using the one-way ANOVA with Dunnett’s multiple comparison tests. p-values were adjusted using the false discovery rate (FDR) method. For glycomic analysis, the relative intensities of glycan structures with unique m/z, observed in their sodiated forms, were calculated for each replicate. Normality of each protein were assessed with Shapiro-Wilk test. The statistical significance for glycan structures between groups was analysed by t-test, ANOVA and TukeyHSD *post-hoc* test. Shapiro-Wilk test, ANOVA and TukeyHSD *post-hoc* test were performed in R (45), version 4.3.2. Differences were considered statistically significant if ANOVA p-value/FDR ≤ 0.05 or marginally significant if 0.05 < ANOVA p-value/FDR ≤ 0.1.

Transcriptomic analysis of TNF-α stimulated fibroblasts and non-stimulated fibroblasts (PMM2-CDG and control) was conducted using R (v.4.1.1) (45). Quality controlled read counts were assembled on a matrix and the low-quality reads counts were filtered. DEGs between TNF-α stimulated and non-stimulated conditions of the PMM2-CDG and control samples were obtained using the edgeR package (v.3.36) with a cut-off of FDR ≤ 0.05. Over-representation analysis was performed using ToppFun functionality of ToppGene Suite (v.31) platform (46) to identify enriched pathways and Gene Ontology (GO) biological processes, with FDR-adjusted p ≤ 0.05 as the cut-off. Fold enrichment was calculated by the proportion of DEGs present in the GO terms relative to all annotated genes present in that term. Enriched GO terms were further visualised using Reactome (47). Graphical representation – including principal component (PC) analysis, volcano plots, scatter plots, heatmaps, interaction plots, and functional enrichment plots – were generated in R, and all analysis scripts are available online (48).

## 3 Results

### 3.1 TNFR1 in PMM2-CDG fibroblasts upon TNF-α stimulation

**Figure 1:**
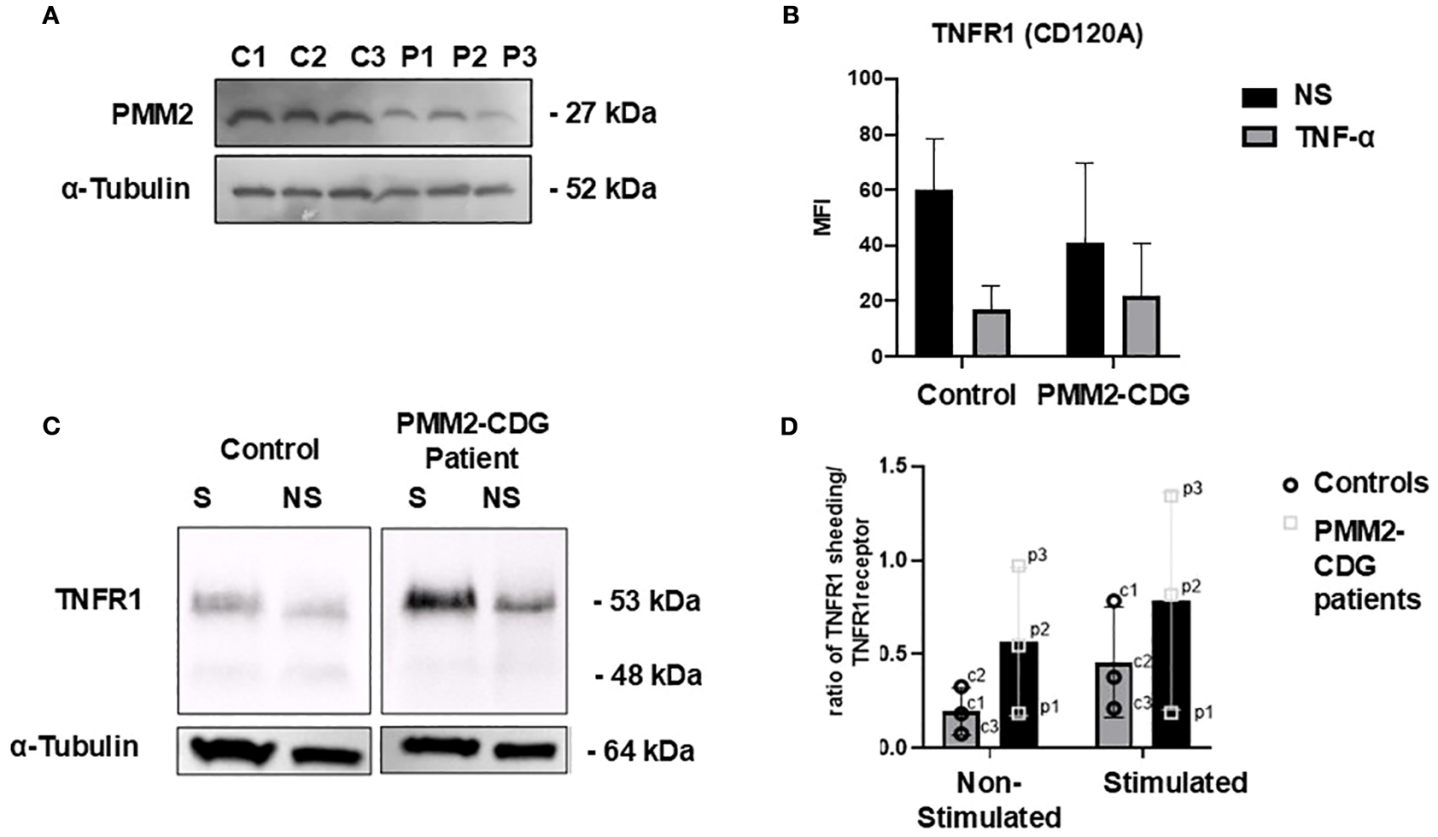
TNFR cell phenotyping in stimulated and non-stimulated PMM2-CDG and control fibroblasts. Fibroblasts were stimulated with 10 ng/ml of TNF-α for 24h and analysed using different techniques. **(A)** Western blot analysis of the PMM2 protein in PMM2-CDG and WT skin fibroblasts. Immunoblotting of cell lysates from three PMM2-CDG patients and three controls fibroblasts was performed using anti-PMM2 (Proteintech) at 1:1000. Mouse monoclonal anti-α-tubulin staining was performed as loading control; **(B)** Flow cytometry was performed for TNFR1 cell surface staining and **(C)** Western blot of cell lysates was performed for TNFR1 immunoblotting to access the overall expression of TNFR1. Staining with α-tubulin was used as loading control. The patient and control samples shown correspond to P1 and C1, respectively, as listed in **Table 1**. **(D)** Ratio of TNFRI shedding (soluble form) to membrane bound TNFR1 in non-stimulated and stimulated conditions in fibroblasts from PMM2 patients and healthy controls. Each data point represents an individual cell line (**Table 1**). p1: patient 1, p2: patient 2, p3: patient 3, c1: control 1, c2: control 2, c3: control 3. Data were confirmed for a normal distribution using a Normal Q-Q plot and Shapiro- Wilk test. Statistical comparisons were performed using an unpaired t-test; no statistically significant differences were found. Bars represent the mean ± standard deviation.

Skin fibroblasts from three PMM2–CDG patients (4.3 years average age) and three controls (5 years average age) were used to investigate TNFR1 expression. All patient cells exhibited reduced PMM2 expression (**Figure 1A**). The most frequently reported variant, p.R141H (49), was also included. We performed flow cytometry and Western blot of whole cell lysates analysis to assess whether PMM2 defects affected TNFR1 levels at cell surface or globally. We observed a decrease in TNFR1 surface expression after TNF–α stimulation with no statistical difference between WT and PMM2–CDG fibroblast, before or after stimulation (**Figure 1B**). However, the analysis of TNFR1 expression in whole cell lysates (**Figure 1C**) revealed two differentially expressed bands. A more intense ~63 kDa band which is intensified upon stimulation, especially in the patient sample compared to all other conditions. In addition, the 48 kDa band, corresponding to the shedding of the receptor, is less intense in patients. Taking into consideration the ~63 kDa band, these results also suggest that TNF–α stimulation increases its glycosylation in control and PMM2–CDG fibroblasts, as per the appearance of slightly higher molecular weight of this band in the stimulated conditions (**Figure 1C**). When analysed the ratio between the shedding band (48 kDa) and the TNFRI band (63 kDa) (**Figure 1D**), we observed a difference between groups. While in non–stimulated conditions, both patients and controls have low and comparable ratios, upon stimulation, the patient group displays a wider spread of values, with two samples showing markedly elevated ratios (patients with severe mutation R141H).

### 3.2 N-glycophenotype of PMM2-CDG is altered upon TNF-α stimulation

**Figure 2:**
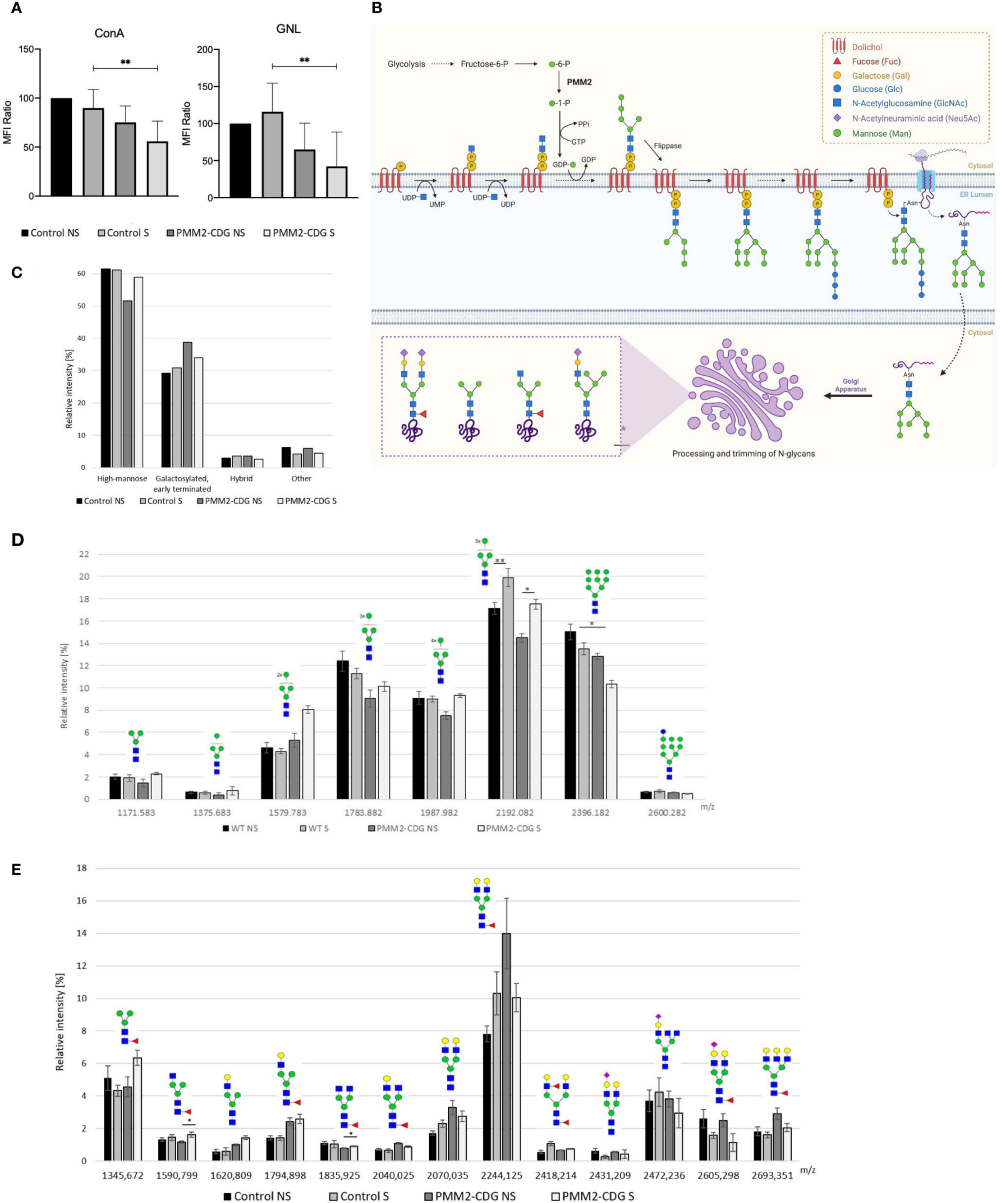
N-glycoprofiling in stimulated and non-stimulated PMM2-CDG and control fibroblasts. PMM2-CDG and control fibroblasts were stimulated (S) or non-stimulated (NS) with TNF-α (10 ng/ml) for 24h and analysed using different techniques. **(A)** Lectin staining was performed resorting to the ConA and GNL lectins and analysed by flow cytometry. Data are represented as the mean ± SD of normalized mean fluorescence intensity (MFI) values to the WT non-stimulated group (n = 3); **(B)** N-glycosylation biosynthetic pathways with emphasis on the N-glycan structures identified in this study. N-glycan synthesis begins in the cytosolic side of the endoplasmic reticulum (ER) with the attachment of N-acetylglucosamine (GlcNAc) to dolichol monophosphate. This is followed by the addition of one GlcNAc and five mannose residues, using a nucleotide-activated sugar, guanosine diphosphate-mannose (GDP-Man), as the donor. The PMM2 enzyme plays a critical role in the in GDP-Man biosynthesis, by converting mannose-1-phosphate in mannose-6-phosphate. The lipid precursor is then translocated by a flippase to the lumen of the ER, where it undergoes further elongation. Once the oligosaccharide (Glc3Man9GlcNAc2) is completed, it is transferred to an asparagine residue of the nascent protein. The processing of N-glycans is initiated in the ER and continues in the Golgi apparatus, resulting in the formation of various N-glycans forms, such as high mannose, galactosylated, early terminated, fucosylated agalactosylated bi-antennary and hybrid N-glycans. Image created using BioRender (www.biorender.com); **(C)** Semi-quantitative representation of four main N-glycan groups based on their type (high mannose; galactosylated and early terminated; hybrid; and other glycans) determined by the sum of relative intensities of individual structures by MALDI-TOF mass spectrometry; **(D)** relative distribution of individual high mannose N-glycans (Man3GlcNAc2-Man10GlcNAc2) determined by MALDI-TOF mass spectrometry; and **(E)** relative distribution of individual galactosylated and early terminated N-glycans determined by MALDI-TOF mass spectrometry. Data from mass spectrometry analysis is based on four technical replicates, except stimulated PMM2-CDG fibroblasts that were measured in triplicate due to limited sample amount. p < 0.05 (*), p < 0.01 (**). green circle – mannose, yellow circle – galactose, blue square – N-acetylglucosamine, red triangle – fucose, purple diamond – sialic acid.

N-glycosylated profile of cell surface and whole fibroblasts was performed using lectin staining and MALDI-MS analysis. For lectin staining we used lectins able to detect N-glycans expressed in TNFR1 (34). Namely, ConA that bind to high mannose N-glycan structures, and selectively high-mannose terminated glycans and early terminated biantennary complex N-glycans and GNL lectin more selective for alpha1–6 and alpha1–3 mannose-terminated N-glycans. Results show that PMM2-CDG fibroblasts reveal the expected decrease in N-glycosylation under normal conditions (**Figure 2A**). However, after TNF-α stimulation, the levels of all N-glycan structures significantly decreased in PMM2-CDG, as shown by ConA staining (p = 0.009) and GNL staining (p = 0.008), but not in control cells (**Figure 2A**).

To gain a better understanding of the nature of the altered N-glycans along the N-glycosylation pathway (**Figure 2B**), we performed MALDI-MS analysis, which identified 47 N-glycans (**Supplementary Figure 2**). Of all N-glycans identified, high mannose N-glycans (Man3–Man9GlcNAc2) represented the most abundant group, comprising over 50% (**Figure 2C**). Following TNF-α stimulation, the abundance of Man8GlcNAc2 (m/z 2192.08) increased significantly in both control and PMM2-CDG samples (ANOVA, p = 0.00042). *Post-hoc* Tukey HSD analysis revealed significant differences between unstimulated and stimulated controls (p = 0.004) and between unstimulated and stimulated PMM2-CDG samples (p = 0.028). Conversely, it resulted in a consistent increase of lower high mannose glycans (Man3-Man8GlcNAc2) that serve as precursors of Man9GlcNAc2 in the N-glycan biosynthesis pathway or as products of Man9GlcNAc2 degradation in the cis-Golgi apparatus. Following TNF-α stimulation, the abundance of Man8GlcNAc2 (m/z 2192.08) increased significantly in both control and PMM2-CDG (ANOVA p = 0.00042, TukeyHSD *post-hoc* p = 0.004 and p=0. 0.028, respectively) (**Figure 2D**). The increase was more pronounced in PMM2-CDG (21% compared to 16% in control), while the levels of Man3-Man7GlcNAc2 glycans remained unchanged. The second largest identified group included galactosylated and early terminated N-glycans, among which the relative intensity of fucosylated agalactosylated bi-antennary N-glycans, terminated by one (m/z 1590.80) or two N-acetylglucosamines (m/z 1835.93), was significantly increased upon TNF-α stimulation of PMM2-CDG cells (p=0.029 and 0.019, respectively), in contrast to controls (**Figure 2E**). Hybrid N-glycans, although <4% of the total, showed an increase in controls and a decrease in PMM2-CDG cells upon TNF-α stimulation (**Supplementary Figure S2**). These results indicate alterations in N-glycan processing within PMM2-CDG, especially related to decreased high mannose glycans and increased of lower mannose glycans, which are intensified upon TNF-α stimulation.

### 3.3 Transcriptional profiling shows altered inflammatory and immune pathways in PMM2-CDG fibroblasts

To further investigate the molecular basis, we performed transcriptomic profiling of patient-derived fibroblasts under basal conditions and following TNF-α stimulation. Principal component analysis (PCA) of the transcriptome data revealed four distinct clusters, reflecting both inflammatory status (stimulated *vs*. non-stimulated) and genotype (**Supplementary Figure S3A**). PC1 separated stimulated and non-stimulated samples, while PC2 separated most PMM2-CDG from controls, confirming genotype-dependent transcriptional differences associated with PMM2-CDG. Notably, samples from the patient with p.L32R/p.F157S genotype clustered closer to control samples (**Supplementary Figure S3A**), whereas those from patients with the variant p.R141H (p.R141H/p.N216I and p.R141H/p.E139K) resulted in a clearer separation (**Supplementary Figure S3B**).

**Figure 3:**
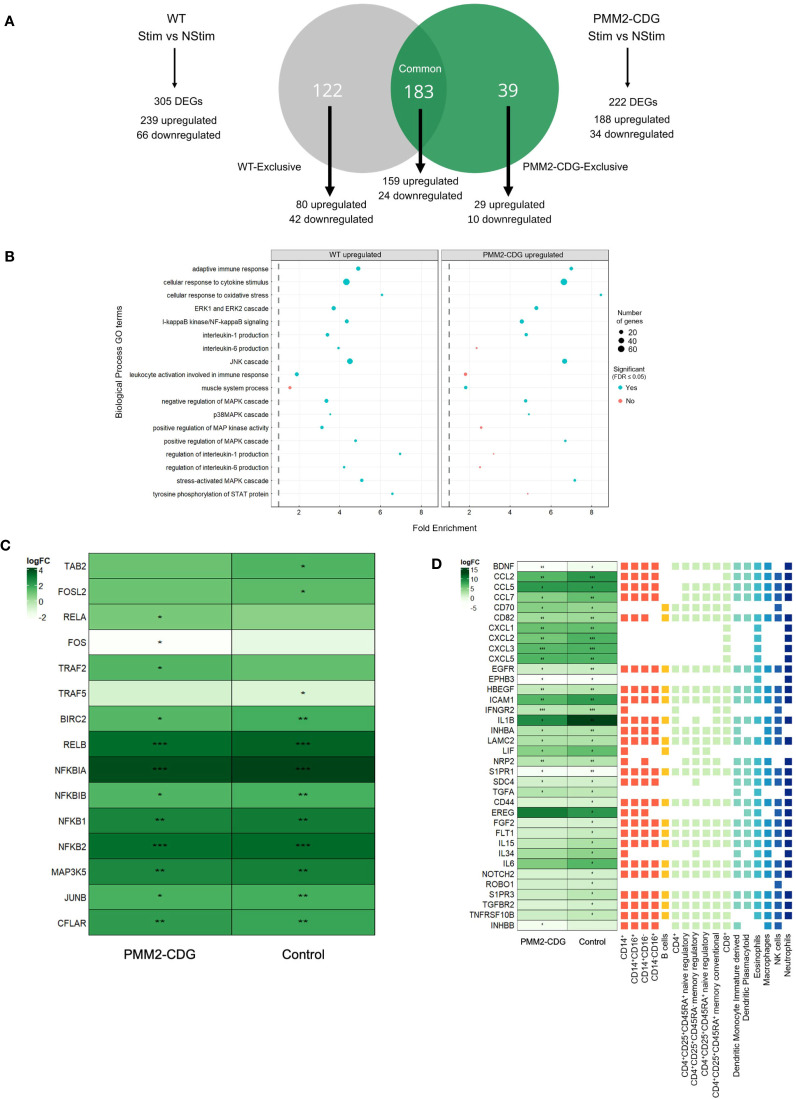
Differential gene expression and functional annotation analysis of TNF-α stimulated and non-stimulated samples of control and PMM2-CDG samples. **(A)** Venn diagram of control *vs* PMM2-CDG DEGs upon TNF-α stimulus. A total of 305 (239 upregulated and 66 downregulated) and 222 DEGs (188 upregulated and 34 downregulated) were identified in the control and PMM2-CDG sample groups, respectively. These DEGs can be classified into three distinct groups: Control-exclusive, Common, and PMM2-CDG-exclusive. There is a total of 122 DEGs in the control-exclusive group, of which 80 are upregulated and 42 are downregulated. The Common group contains 183 DEGs, 159 upregulated and 24 downregulated. The PMM2-CDG-exclusive group comprises 39 DEGs, of which 29 are upregulated and 10 are downregulated. **(B)** Fold enrichment plot from the upregulated control and PMM2-CDG sample groups of the GO terms within the Biological Process domain. Blue dots represent the GO terms which are statistically significant, while the red dots comprise the GO terms which are not statistically significant. The size of the dots comprehends the number of DEGs which are inserted in the pathway of the GO term. **(C)** Heatmap of the log2FC of signalling, adaptor and regulatory proteins involved in TNFR1 signalling. **(D)** Log2 Fold-Change **(FC)** of differently expressed receptors and ligands upon TNF-α stimulus in PMM2-CDG and control skin fibroblasts, annotated with a prediction of cell–cell interactions with immune cells. These Log2FC are statistically significant, with False Discovery Rate (FDR) < 0.05 (*), FDR < 0.01 (**) or FDR < 0.001 (***). The receptor and ligands represented include common DEGs between PMM2-CDG and control samples (significant in both conditions), control-exclusive (only significant in control) and PMM2-CDG DEGs (only significant in PMM2) upon TNF-α stimulation. Different colours were used to distinguish between immune cell types.

Differential expression analysis of TNF-α responsive genes showed 305 DEGs in control cells (239 upregulated, 66 downregulated) and 222 in PMM2-CDG cells (188 upregulated, 34 downregulated) (**Supplementary Table S3**, **Supplementary Figure S4**). These DEGs were categorized into common, control-exclusive, and PMM2-CDG-exclusive, illustrating distinct transcriptional responses to TNF-α (**Figure 3A**) and revealing key transcriptional differences. Functional enrichment analysis revealed that PMM2-CDG cells exhibited stronger enrichment for GO terms associated with MAPK, JNK, ERK1/2 and p38 MAPK cascades (**Figure 3B**), **Supplementary Table S4**). In contrast, control samples showed enrichment for GO terms, such as IL-6 production and leukocyte activation, which were absent in PMM2-CDG group (**Figure 3B**), **Supplementary Table S5**). PMM2-CDG-exclusive GO terms point to a dysregulation in JNK activity, serine phosphorylation of Signal Transducers and Activators of Transcription (STAT) proteins, and positive regulation of the p38 MAPK cascade (**Figure 3B**), **Supplementary Table S6**), suggesting altered intracellular signalling downstream of TNFR1.

To further investigate TNFR1 signalling, we analysed the expression of key adaptor and regulatory proteins. Upon TNF-α stimulation, TRAF5, TAB2, and Fos-Like 2 (FOSL2) expression increased in control fibroblasts but not in PMM2-CDG. In contrast, TRAF2, v-rel Avian Reticuloendotheliosis Viral Oncogene Homolog A (RELA, also known as p65), and FOS expression upregulated in PMM2-CDG but remained unchanged in controls (**Figure 3C**), indicating a divergent activation pattern of TNFR1 downstream signalling components.

We also examined, based on DEG analysis, potential interactions between fibroblast and immune cells upon TNF-α stimulus. This analysis revealed thirty-seven genes with potential roles in influencing immune cell interactions differentially expressed (**Figure 3D**). PMM2-CDG fibroblasts exhibited reduced expression of IL-6 and CCL2, suggesting a diminished capacity to activate monocytes and macrophages. Additionally, several DEGs suggest these cells showed impaired potential to support dendritic cell maturation and to modulate regulatory and memory T cell responses. Expression of CCL5, which influences interactions with various immune cells, was also reduced, further supporting a compromised ability to initiate effective immune activation (**Figure 3D**). While these findings suggest genotype-related transcriptomic differences, they should be interpreted as hypothesis-generating due to the limited number of patient-derived samples.

### 3.4 TNF-α downstream signalling and cytokine expression are deregulated in PMM2-CDG fibroblasts

**Figure 4:**
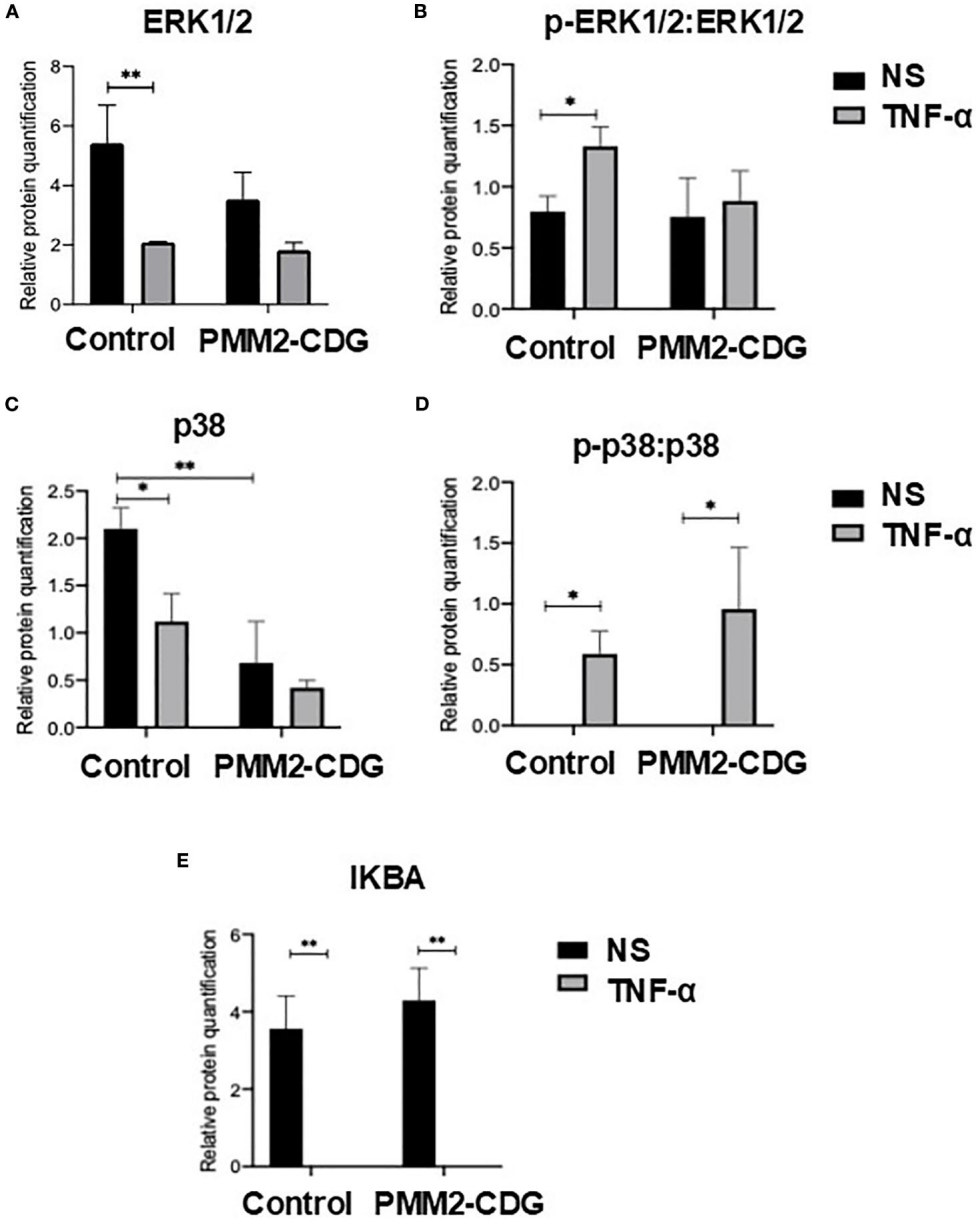
Expression of key TNFR1 signalling proteins. Relative protein quantification of cell signalling proteins, namely ERK1/2 **(A)** and p38 **(C)**, and the ratio of its phosphorylated forms [p-ERK1/2 **(B)** and p-p38 **(D)**] over ERK1/2 and p38, and relative quantification of IκBα **(E)**. Total proteins were obtained from non-stimulated (NS) and TNF-α stimulated fibroblasts (t = 30 min) samples and the expression level of each protein was identified by western blot. The detection of α-tubulin was for band normalization (housekeeping protein normalization method). Statistically significant values were accessed by a one-way ANOVA with Sidak’s multiple comparisons [p < 0.05 (*), p < 0.01 (**)].

To assess the functional consequences of TNFR1 signalling in PMM2-CDG, we analysed the expression and activation of key downstream signalling proteins and cytokine in fibroblasts stimulated with TNF-α. Western blot results indicated that TNF-α stimulation induced a significant decrease in the expression levels of both ERK1/2 and p38 in the controls (p=0.006 and p=0.02, respectively), but did not have a significant effect in PMM2-CDG fibroblasts (**Figure 4A, C**), respectively). The basal levels of p38 MAPK were significantly lower in PMM2-CDG than in the controls (**Figure 4C**). Phosphorylation levels of ERK1/2 and p38, normalized to total protein, increased significantly in control fibroblasts following TNF-α stimulation (**Figures 4B, D**), indicating proper activation of these signalling pathways. In contrast, PMM2-CDG fibroblasts failed to upregulate p-ERK1/2, suggesting impaired signal transduction downstream of TNFR1. NF-κB activation, assessed indirectly via inhibitor of nuclear factor kappa-B alpha (IκBα) levels, showed no significant differences between control and PMM2-CDG fibroblasts (**Figure 4E**), indicating that this pathway may be less affected or regulated differently in the context of glycosylation defects. Additionally, JNK2 protein was nearly undetectable in PMM2-CDG fibroblasts (**Supplementary Figure S5**), further supporting disruption of MAPK signalling.

**Figure 5:**
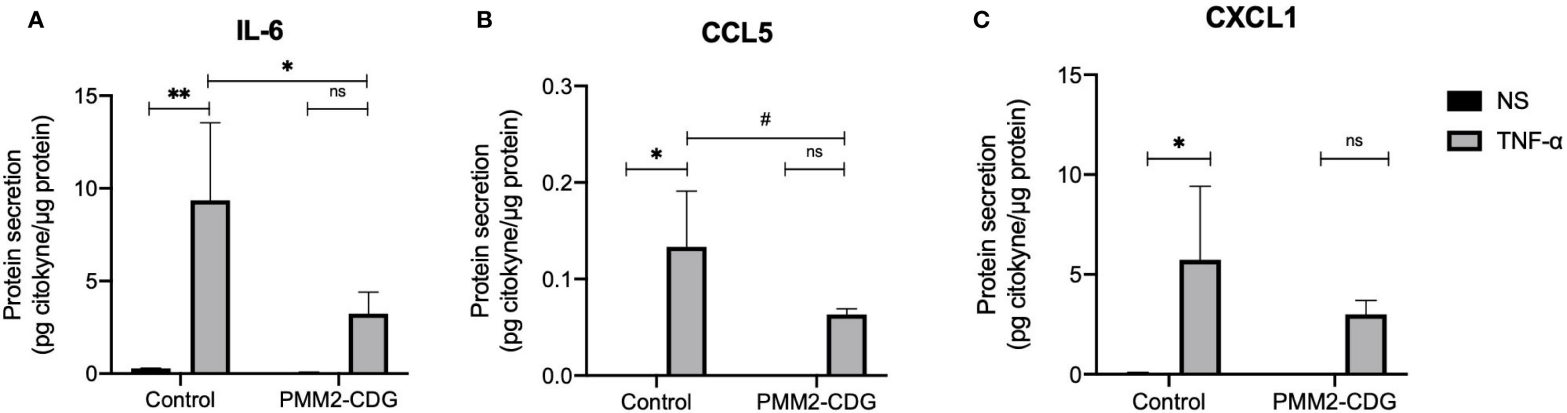
Predicted interactions between fibroblasts and immune cells based on the identified DEGs and quantification of secreted ligands. Cytokine production of NS and TNF-α stimulated samples (t = 24 h) measured by ELISA (IL-6) **(A)** or by a Mix and Match LEGENDplex kit [CCL5 **(B)** and CXCL1 **(C)**]. Data are represented as the mean ± SD (n = 3). Statistically significant values were accessed by a one-way ANOVA with Dunnett’s multiple comparisons (p < 0.05 (*), p < 0.01 (**), 0.05 < p ≤ 0.1 (#). Normalized values were obtained by dividing the concentration of cytokines (pg/mL) by the total protein concentration (mg/mL).

To evaluate the functional output of these signalling alterations, we measured cytokine secretion in response to TNF-α. In control fibroblasts, TNF-α stimulation significantly increased IL-6, CCL5, and CXCL1 secretion (p = 0.004, p = 0.002, and p = 0.024, respectively; **Figures 5A–C**). In contrast, this upregulation was absent in PMM2-CDG fibroblasts, consistent with the observed defects in MAPK pathway activation. We also examined additional receptors and ligands encoded by DEGs identified above. IL-1β and IL-15 showed no detectable protein expression, while CD44 and TNF Receptor Superfamily Member 10B (TNFSF10B), did not exhibit significant differences between control and PMM2-CDG samples (data not shown).

## 4 Discussion

**Figure 6:**
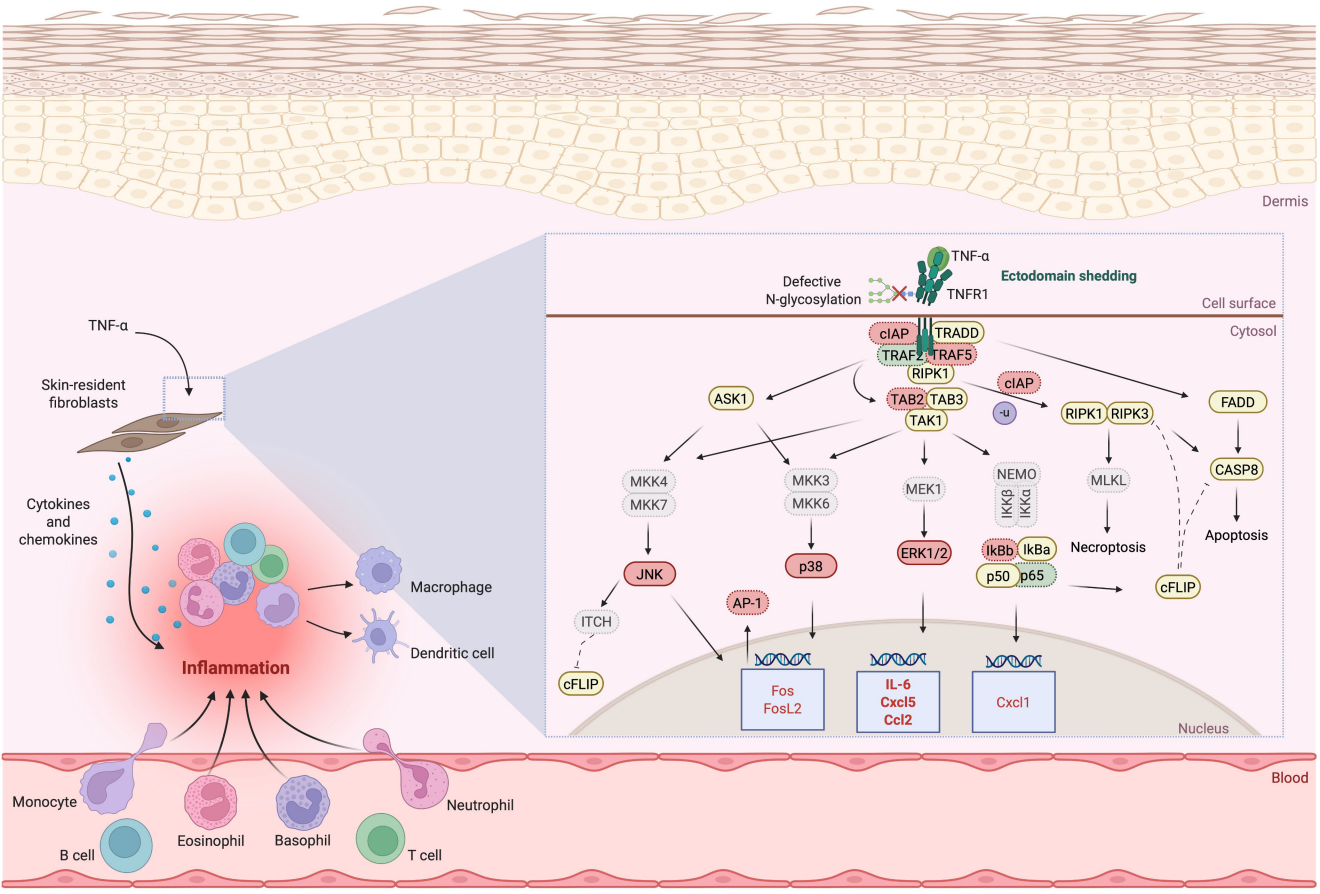
Illustration of the proposed deregulated signalling, adaptor and regulatory proteins involved in TNFR1 signalling due to defective N-glycosylation and TNFR1 shedding. Downregulated expression is shown in red, upregulated expression is shown in green. The proteins whose expression does not differ between control and PMM2-CDG or where is no indication of their expression are highlighted in yellow. For the proteins where differences were identified or inferred (down or upregulated in PMM2-CDG), when the levels of expression are indicated by only gene expression levels, these are outlines by a dotted border; when the expression levels were identified by protein expression, these are outlined by a continuous border. Expression levels confirmed by both gene and protein levels are in bold. AP-1, Activator protein 1; ASK1, Apoptosis-stimulated kinase 1; CASP8, caspase 8; CCL, Chemokine C-C motif ligand; cFLIP, cellular FLICE inhibitory protein; cIAP, Cellular inhibitor of apoptosis protein; CXCL, C-X-C motif chemokine ligand; ERK, Extracellular signal-regulated kinase; FADD, FAS-associated death domain; FOS(L), Fos Proto-Oncogene (like); IKKα/β, Inhibitory kappa B kinases alpha/beta; IL, Interleukin; ITCH, ubiquitin E3 ligase ITCH, also named atrophin-1 interacting protein 4; IκBα/β, Nuclear factor kappa-light-chain-enhancer of activated B cells inhibitor alpha (/beta); JNK, c-Jun N-terminal kinase; MEK/MKK, Mitogen-activated protein kinase kinase; MLKL, Mixed lineage kinase domain-like protein; NEMO, Nuclear factor kappa-light-chain-enhancer of activated B cells essential modulator; RIPK, Receptor-interacting serine/threonine-protein kinase; TAB, TAK1-binding protein; TAK, Transforming growth factor-β (TGFβ)-activated kinase; TNF(R), Tumour necrosis factor (receptor); TRADD, TNF receptor type 1-associated death domain; TRAF, TNF receptor associated factor. Several abbreviations used in the figure are not described in the text and are shown in grey. Image created using BioRender (www.biorender.com).

The clinical complexity of PMM2-CDG, particularly its immune-related manifestations, highlights the intricate role of glycans in immune regulation (26). Although immune-related manifestations are common in PMM2-CDG patients, detailed molecular studies remain scarce, primarily due to the rarity of the disease and resulting limited patient-derived samples. Previous studies have indicated that PMM2-CDG patients exhibit a differential inflammatory response contributing to disease burden (19, 50). Glycosylation pathways are complex and module immune signalling by influencing receptor ligand interactions, antigen presentation, and immune cell differentiation (51, 52). In CDG, glycan disruptions can lead to immune dysregulation and disease pathogenesis (19). In this study, we investigated how defective glycosylation in PMM2-CDG alters immune function, focusing specifically on TNF-α mediated signalling using patient-derived fibroblasts as a model system (**Figure 6**).

For the first time, we report structural abnormalities in TNFR1 in PMM2-CDG fibroblasts, along with reduced cell surface expression following TNF-α stimulation (**Figure 1A**). As expected from receptor internalisation mechanisms (51), TNFR1 expression increased in total cellular extracts after stimulation. Interestingly, a distinct ~48 kDa TNFR1 fragment (**Figure 1B**) observed upon stimulation, consistent with receptor ectodomain shedding mediated by TNF-α converting enzyme (TACE) was differently expressed (53). In fact, in PMM2-CDG, TNFR1 shedding was reduced, while the levels of membrane-bound TNFR1 are increased (**Figures 1C, D**). This indicates a decrease in the catalytic activity of TACE enzyme, which is responsible for cleaving and releasing TNFR1 from the cell surface, in agreement with the impact of glycosylation on the TACE activity (54). Impaired TACE results in more TNFR1 receptors remaining on the cell membrane, potentially making cells more sensitive to TNF-α signalling. At the same time, the reduced presence of the soluble form limits the ability to neutralise circulating TNF-α. These alterations may contribute to a heightened proinflammatory state and the dysregulation of the immune response (55–60). Elevated TNF-α levels have also been shown to increase blood-brain barrier permeability, exacerbating neuroinflammation and tissue damage during the acute phase of a stroke. However, TNF-α expression has not yet been investigated in the context of PMM2-CDG (61, 62). In these patients, stroke like episodes– often triggered by infection – are a significant cause of morbidity and associated with long-term decline in quality of life (63). Understanding how TNF-α signalling is altered in PMM2-CDG and its interplay with infections is crucial for advancing clinical care (63). Our findings suggest that defective N-glycosylation may structurally impact TNFR1 upon TNF-α engagement, contributing to abnormal receptor processing and signalling, which is in agreement with previous studies. In fact, other showed that N-glycosylation of TNFR1 can enhance its ability to bind to TNF-α, promoting a TNF-α autocrine feedback loop, that amplifies the inflammatory process, while elimination of N-glycosylation suppressed ligand-binding affinity (34). Our data also provides additional evidence that, less severe mutations like the one from patient 1, cluster closer to the values from control samples than the more severe mutations (patients 2 and 3). This suggests that the severity of the mutation may influence the extent of TNFR1 shedding dysregulation. Milder mutations appear to preserve TACE function, potentially resulting in a less severe proinflammatory profile.

Inflammation has been associated with alterations in glycosylation, which can vary depending on the cell type and environment (64–66). In line with this, the surface N-glycoprofile of PMM2-CDG fibroblasts was significantly altered upon TNF-α stimulation (**Figure 2**). The most significant changes occurred in high-mannose N−glycans, consistent with previous observations in other PMM2-CDG cells (67). Specifically, we report for the first time a reduction in Man5-8GlcNAc2 levels in PMM2-CDG fibroblasts, which was reversed by TNF-α stimulation. These differences may arise from altered impaired N-glycan synthesis in the endoplasmic reticulum, due to a limited supply of Man-1-P necessary for GDP-mannose synthesis (68) or from altered N-glycosylation processing in the Golgi apparatus, where mannosidases trim N-glycans. In contrast to Man5-8GlcNAc2 N-glycans, the relative abundance of Man9GlcNAc2 decreased following TNF-α stimulation in both control and PMM2-CDG fibroblasts. These results suggest that TNF-α stimulation exerts a direct influence on the remodelling and processing of mannosylated glycans. Given that TNFR1 is known to be N-glycosylated with high-mannose and complex type structures, the glycan structures detected are likely attributable, at least in part, to TNFR1 (34). Altered N-glycosylation may thus contribute to the increase of inflammatory responses commonly observed in PMM2-CDG (64).

Supporting the link between glycosylation and inflammatory signalling, our data revealed that fibroblasts from patients carrying the p.R141H variant exhibited a distinct transcriptional signature upon TNF-α stimulation, with altered expression of genes involved in MAPK and NF-κB pathways. This aligns with clinical observations that p.R141H is the most severe heterozygous mutation (49). These findings also align with our previous ImmunoCDGQ e-questionnaire, where 41 out of 122 PMM2-CDG patients, carried the p.R141H variant and reported more immune-related manifestations, including frequent or severe infections, unexplained fever episodes and altered vaccination responses (19). Furthermore, several of these individuals experienced septic episodes (69), reinforcing the notion that genotype influences immune vulnerability. When comparing genotypes in more detail, patients with the p.R141H/p.N216I and p.R141H/p.E139K showed clearer transcriptomic distinctions from controls (**Supplementary Figure S3B**), suggesting a stronger impact of the p.R141H variant on the cellular transcriptional profile. In addition, the patient with p.L32R/p.F157S harbours two folding-defective alleles, which are expected to yield some residual enzyme function, in contrast the patient with p.R141H/p.N216I combines a catalytic-null mutation with a folding defective allele resulting in lower residual activity, while p.R141H/p.E139K carries a catalytic-null mutation together with a mutation that leads to high residual PMM2 activity and less thermolability (**Table 1**) (35). This highlights how compound heterozygous mutations can differentially alter enzymatic function and contribute to phenotypic variability. Taken together, these results suggest that immunological dysfunction in PMM2-CDG is not uniform but modulated by genetic background, with the p.R141H variant contributing to more pronounced immune dysregulation.

To further understand the impact of TNF-α signalling in PMM2-CDG, we analysed the DEGs following stimulation and identified three striking differences between control and PMM2-CDG fibroblasts: 1) the number of TNF-α-responsive genes; 2) exclusive genes up or downregulated in one of the groups and; 3) differential magnitudes of expression of genes that respond in both groups (**Figure 3A**). These differences indicate gene regulation defects upon inflammatory stimulus which translate into functional disparities. Enrichment analysis (**Figure 3B**) confirmed that TNF-α activated NF-κB signalling and several MAPK cascades (p38, JNK, and ERK1/2) which agree with known standard pathways associated with the activation and regulation of various proinflammatory cytokines (70, 71). Consistently, GO term enrichment showed higher fold enrichment for MAPK, JNK, ERK1/2, and p38 MAPK cascades in PMM2-CDG samples and indicated that these interconnected pathways are dysregulated in PMM2-CDG. Gene expression analysis further revealed that TRAF5, TAB2, and FOSL2 were upregulated in control fibroblasts but not in PMM2-CDG after TNF-α stimulation. In contrast, TRAF2, RELA, and FOS were upregulated in PMM2-CDG but remained unchanged in controls (**Figure 3C**). These results indicate a possible deregulation of TNFR1-mediated signalling via the TRADD–TRAF–TAB2 axis, particularly affecting the ERK1/2 and p38 MAPK pathways in PMM2-CDG. Accordingly, PMM2-CDG fibroblasts produced lower levels of IL-6 and CCL5 after TNF-α stimulation (**Figure 5**), both of which are crucial for immunomodulation and chemotaxis (72, 73). This observation is supported by studies in a N-glycosylation deficient mouse model showing impaired neutrophil extravasation during inflammation (74). These deficiencies—particularly the reduced IL-6 levels—may impair interactions with a variety of immune cells, including monocytes, T cells, B cells, dendritic cells, eosinophils, macrophages, natural killer (NK) cells, and neutrophils. Similarly, diminished CCL5 expression could affect communication with the same cell types, except B cells and CD8^+^ T cells (**Figure 3D**). The decreased levels of these cytokines might partially explain the recurrent and severe infections of PMM2-CDG patients (19).

To further explore the functional basis of these gene expression defects, we examined the activation state of major TNFR1 downstream signalling proteins. PMM2-CDG fibroblasts present defective phosphorylation of p38 and JNK proteins, which aligns with the reduced cytokine expression and altered gene and transcription factors regulated by these pathways (**Figure 4**) (70, 75, 76). Notably, FOS and FOSL2, which codify the Fos protein, and are regulated by ERK1/2 (70, 77), were significantly downregulated in PMM2-CDG. Additionally, JNK downregulation limits Jun activation (78), which suggest that PMM2-CDG have impaired activating protein-1(AP-1) complex formation (composed of Fos and Jun proteins), critical for proper cytokine expression and inflammatory regulation (79). While we observed deregulated expression of several NF-κB-target genes, there was no significant alteration in NF-κB activation, this means that patient cells are still capable of initiating the inflammatory response. Yet, due to defective MAPK, JNK, p38 signalling, the response cannot be properly amplified and regulated. It has been shown that when p38 is inhibited NF-κB transcriptional activity will be blocked, even though DNA binding and nuclear translocation still occur. MAPK p38 regulates transcriptional activity not by altering NF-κB activation, but by controlling the activity of coactivator p300. p300 promotes acetylation of p65 at K310, allowing proper transcription of proinflammatory genes, so if this coactivator is not properly working NF-κB target genes will not be properly express (80).This suggests that TNFR1 glycosylation defects do not globally impair receptor signalling but rather selectively affect branches requiring specific adaptor recruitment or receptor clustering, which are more sensitive to glycosylation status. This imbalance could contribute to the paradoxical phenotype observed by others in PMM2-CDG patients: elevated systemic inflammatory markers during infections but impaired local immune responses, leading to recurrent and severe infections (63, 16–18, 29). Further studies are still needed to clarify the complete signalling dysregulation, including NF-κB complexes formation and its transcriptional activity in the context of PMM2-CDG.

Despite the robustness of our findings, this study has some limitations. While fibroblasts are not classical immune cells and this may constrain the direct immunological relevance of our results, they are increasingly recognized as active modulators of immune response and thus provide a valuable model to investigate inflammatory mechanisms (81). Regarding the western blot findings on TNFR1, they require further exploration using complementary techniques, like structural and biochemical analysis to fully characterize the receptor. Additionally, the use of 3′-end RNA sequencing may underestimate transcript diversity and low-abundance gene expression. However, as far as we know, this is the first study aimed at systemically dissect the underlying impaired mechanisms in the immune response of PMM2-CDG patients in response to a proinflammatory stimulus, offering foundational insights into the disease-associated immune dysregulation. PMM2-CDG is an ultra-rare disease, with a prevalence estimated at 1 in 63,694 (82). As such, the limited availability of patient-derived samples is a well-recognized challenge in this field. Studies in rare diseases often rely on small cohorts, and findings are considered by regulatory agencies mostly hypothesis-generating rather than definitive (83). Nevertheless, our findings provide valuable insights and datasets grounding the foundation for future studies as more patient-derived material becomes accessible.

In conclusion, our findings demonstrate that defective glycosylation in PMM2-CDG alters TNFR1 structure and signalling, leading to defective activation of key inflammatory pathways and reduced cytokine secretion. These insights not only advance our understanding of PMM2-CDG immunopathology but also identify potential therapeutic targets, such as TNFR1 modulation or glycosylation restoration strategies. This work lays the foundation for future studies exploring targeted immunotherapies in CDG and related disorders.

## Data Availability

The datasets presented in this study can be found in online repositories. The names of the repository/repositories and accession number(s) can be found in the article/**Supplementary Material.**

## Supplementary material

Supplementary Table 1 Details of reagents, instruments, software and other materials used in the methodology of this work.

Supplementary Table 2 Cell-cell predicted interactions based the DEG of WT and PMM2-CDG TNF-α stimulated fibroblasts and on ligand/receptor gene expressions

Supplementary Table 3 Differential expressed genes of WT and PMM2-CDG skin fibroblasts. The differentially expressed genes (DEG) between TNF-α stimulated and non-stimulated PMM2-CDG and WT fibroblasts were obtained using the “edgeR” (v 3.36) package in with a cut-off of False Discovery Rate (FDR) ≤ 0.05)

Supplementary Table 4 Enriched gene ontology (GO) terms common between WT and PMM2-CDG stimulated fibroblast

Supplementary Table 5 Enriched WT-exclusive GO terms

Supplementary Table 6 Enriched PMM2-exclusive GO terms. The GO terms (particularly biological processes, molecular functions, and cellular components) enriched in TNF-α stimulated WT and PMM2-CDG fibroblasts were obtained following an over-representation analysis biological processes using the Toppfun functionality of the Toppgene Suite (v 31) platform. FDR adjusted p ≤ 0.05 was set as the cut-off criteria

## Glossary

AP-1: Activating Protein-1
ASK1: Apoptosis signal-regulating kinase 1
BCA: Bicinchoninic Acid
CASP8: Caspase 8
CCL: Chemokine C-C Motif Ligand
CDG: Congenital Disorders of Glycosylation
cFLIP: cellular FLICE inhibitory protein
cIAP1/2: Cellular Inhibitor of Apoptosis Protein 1 or 2
ConA: Concanavalin A
CXCL: C-X-C Motif Chemokine Ligand
DEG: Differentially Expressed Gene
ELISA: Enzyme-Linked Immunosorbent Assay
ER: Endoplasmic Reticulum
ERK: Extracellular Signal-Regulated Kinase
FADD: FAS-associated Death Domain
FC: Log2 Fold-Change
FDR: False Discovery Rate
FOS/FOSL2: Fos Proto-Oncogene/Fos-Like 2
GDP-Man: Guanosine Diphosphate-Mannose
GlcNAc: N-acetylglucosamine
GNL: Galanthus Nivalis Lectin
GO: Gene Ontology
HKP: Housekeeping Protein
IKK: inhibitory kappa B kinases
IL: Interleukin
ITCH: Itchy E3 Ubiquitin Protein Ligase, also named atrophin-1 interacting protein 4
IκBα/β: Inhibitor of Nuclear Factor Kappa-B Alpha/Beta
JNK: c-Jun N-terminal Kinase
MALDI-MS: Matrix-Assisted Laser Desorption/Ionization Mass Spectrometry
MAPK: Mitogen-Activated Protein Kinase
MEK/MKK: Mitogen-Activated Protein Kinase Kinase
MFI: Mean Fluorescence Intensity
MLKL: Mixed Lineage Kinase Domain-Like Protein
Na_3_VO_4_: Sodium Orthovanadate
NEMO: Nuclear factor kappa-light-chain-enhancer of activated B cells essential modulator
NF-κB: nuclear factor kappa-light-chain-enhancer of activated B cells
NK: Natural Killer
NS: Non-Stimulated
PBS: Phosphate-Buffered Saline
PCA: Principal Component Analysis
PMM2: Phosphomannomutase 2
PMM2-CDG: Phosphomannomutase 2-Congenital Disorder of Glycosylation
PNGase F: Peptide N-Glycosidase F
PVDF: Polyvinylidene Difluoride
RELA: v-rel Avian Reticuloendotheliosis Viral Oncogene Homolog A, also known as Transcription factor p65 or NF-κB p65 subunit
RIPK1: Receptor-Interacting Serine/Threonine-Protein Kinase 1
S: Stimulated
SDS-PAGE: Sodium Dodecyl Sulfate Polyacrylamide Gel Electrophoresis
STAT: Signal Transducer and Activators of Transcription;TAB2/3, TGFβ-activated kinase, TAK-binding proteins 2 and 3
TACE: Tumour Necrosis Factor-Alpha Converting Enzyme
TAK1: Transforming growth factor-β-activated kinase 1
TGFβ: Transforming Growth Factor Beta
TNF: Tumour Necrosis Factor
TNFR1: Tumour Necrosis Factor Receptor 1
TNFSF10B: TNF Receptor Superfamily Member 10B
TRADD: Tumor Necrosis Factor Receptor Type 1-Associated Death Domain
TRAF2/5: TNF Receptor Associated Factor 2 or 5
WT: Wild Type

## References

[1] Parkin J Cohen B. An overview of the immune system. Lancet. (2001) 357:1777–89. doi: 10.1016/S0140-6736(00)04904-7, PMID: 11403834

[2] Silva Z Soares CO Barbosa M Palma AS Marcelo F Videira PA. The role of sialoglycans in modulating dendritic cell function and tumour immunity. Semin Immunol. (2024) 74–75:101900. doi: 10.1016/j.smim.2024.101900, PMID: 39461124

[3] Silva Z Rabaça JA Luz V Lourenço RA Salio M Oliveira AC. New insights into the immunomodulatory potential of sialic acid on monocyte-derived dendritic cells. Cancer Immunology Immunotherapy. (2024) 74:9. doi: 10.1007/s00262-024-03863-7, PMID: 39487861 PMC11531459

[4] Lee HS Qi Y Im W. Effects of N-glycosylation on protein conformation and dynamics: Protein Data Bank analysis and molecular dynamics simulation study. Sci Rep. (2015) 5:1–7. doi: 10.1038/srep08926, PMID: 25748215 PMC4352867

[5] Lyons JJ Milner JD Rosenzweig SD. Glycans instructing immunity: the emerging role of altered glycosylation in clinical immunology. Front Pediatr. (2015) 3:54. doi: 10.3389/FPED.2015.00054, PMID: 26125015 PMC4463932

[6] MuffelsIJJKoziczTPerlsteinEOMoravaE. The therapeutic future for congenital disorders of glycosylation. J Inherit Metab Dis. (2025) 48. doi: 10.1002/jimd.70011, PMID: 40064184

[7] Péanne R de Lonlay P Foulquier F Kornak U Lefeber DJ Morava E. Congenital disorders of glycosylation (CDG): Quo vadis? Eur J Med Genet. (2018) 61:643–63. doi: 10.1016/J.EJMG.2017.10.012, PMID: 29079546

[8] Pascoal C Francisco R Ferro T dos Reis Ferreira V Jaeken J Videira PA. CDG and immune response: From bedside to bench and back. J Inherit Metab Dis. (2020) 43:90–124. doi: 10.1002/JIMD.12126, PMID: 31095764

[9] Verheijen J Wong SY Rowe JH Raymond K Stoddard J Delmonte OM. Defining a new immune deficiency syndrome: MAN2B2-CDG. J Allergy Clin Immunol. (2020) 145:1008. doi: 10.1016/J.JACI.2019.11.016, PMID: 31775018 PMC7062559

[10] Pascoal C Francisco RB Mexia P Pereira BL Granjo P Coelho H. Revisiting the immunopathology of congenital disorders of glycosylation: an updated review. Front Immunol. (2024) 15:1350101. doi: 10.3389/FIMMU.2024.1350101, PMID: 38550576 PMC10972870

[11] Monticelli M Ferro T Jaeken J dos Reis Ferreira V Videira PA. Immunological aspects of congenital disorders of glycosylation (CDG): a review. J Inherit Metab Dis. (2016) 39:765–80. doi: 10.1007/S10545-016-9954-9, PMID: 27393411

[12] Jaeken J Vanderschueren-Lodeweyckx M Casaer P Snoeck L Corbeel L Eggermont E. Familial psychomotor retardation with markedly fluctuating serum prolactin, FSH and GH levels, partial TBG-deficiency, increased serum arylsulphatase A and increased CSF protein: a new syndrome? Pediatr Res. (1980) 14:179–9. doi: 10.1203/00006450-198002000-00117

[13] Altassan R Péanne R Jaeken J Barone R Bidet M Borgel D. International clinical guidelines for the management of phosphomannomutase 2-congenital disorders of glycosylation: Diagnosis, treatment and follow up. J Inherit Metab Dis. (2019) 42:5–28. doi: 10.1002/JIMD.12024, PMID: 30740725

[14] Granjo P Pascoal C Gallego D Francisco R Jaeken J Moors T. Mapping the diagnostic odyssey of congenital disorders of glycosylation (CDG): insights from the community. Orphanet J Rare Dis. (2024) 19:407. doi: 10.1186/s13023-024-03389-2, PMID: 39482754 PMC11529564

[15] Izquierdo-Serra M Martínez-Monseny AF López L Carrillo-García J Edo A Ortigoza-Escobar JD. Stroke-Like Episodes and Cerebellar Syndrome in Phosphomannomutase Deficiency (PMM2-CDG): Evidence for Hypoglycosylation-Driven Channelopathy. Int J Mol Sci. (2018) 19:619. doi: 10.3390/IJMS19020619, PMID: 29470411 PMC5855841

[16] Schiff M Roda C Monin ML Arion A Barth M Bednarek N. Clinical, laboratory and molecular findings and long-term follow-up data in 96 French patients with PMM2-CDG (phosphomannomutase 2-congenital disorder of glycosylation) and review of the literature. J Med Genet. (2017) 54:843–51. doi: 10.1136/JMEDGENET-2017-104903, PMID: 28954837

[17] Blank C Smith LA Hammer DA Fehrenbach M Delisser HM Perez E. Recurrent infections and immunological dysfunction in congenital disorder of glycosylation Ia (CDG Ia). J Inherit Metab Dis. (2006) 29:592. doi: 10.1007/S10545-006-0275-2, PMID: 16826448

[18] Pascoal C Ferreira I Teixeira C Almeida E Slade A Brasil S. Patient reported outcomes for phosphomannomutase 2 congenital disorder of glycosylation (PMM2-CDG): listening to what matters for the patients and health professionals. Orphanet J Rare Dis. (2022) 17:398. doi: 10.1186/s13023-022-02551-y, PMID: 36309700 PMC9618201

[19] Francisco R Pascoal C Marques-da-Silva D Brasil S Pimentel-Santos FM Altassan R. New insights into immunological involvement in congenital disorders of glycosylation (CDG) from a people-centric approach. J Clin Med. (2020) 9:2092. doi: 10.3390/jcm9072092, PMID: 32635232 PMC7408855

[20] Ong BB Gole GA Robertson T McGill J de Lore D Crawford M. Retinal hemorrhages associated with meningitis in a child with a congenital disorder of glycosylation. Forensic Sci Med Pathol. (2009) 5:307–12. doi: 10.1007/S12024-009-9108-6, PMID: 19851897

[21] García-López R de la Morena-Barrio ME Alsina L Pérez-Dueñas B Jaeken J Serrano M. Natural killer cell receptors and cytotoxic activity in phosphomannomutase 2 deficiency (PMM2-CDG). PloS One. (2016) 11. doi: 10.1371/JOURNAL.PONE.0158863, PMID: 27415628 PMC4944953

[22] Monin ML Mignot C De Lonlay P Héron B Masurel A Mathieu-Dramard M. 29 French adult patients with PMM2-congenital disorder of glycosylation: outcome of the classical pediatric phenotype and depiction of a late-onset phenotype. Orphanet J Rare Dis. (2014) 9:207. doi: 10.1186/S13023-014-0207-4, PMID: 25497157 PMC4266234

[23] Van De Kamp JM Lefeber DJ Ruijter GJG Steggerda SJ Den Hollander NS Willems SM. Congenital disorder of glycosylation type Ia presenting with hydrops fetalis. J Med Genet. (2007) 44:277–80. doi: 10.1136/JMG.2006.044735, PMID: 17158594 PMC2598051

[24] De Lonlay P Seta N Barrot S Chabrol B Drouin V Gabriel BM. A broad spectrum of clinical presentations in congenital disorders of glycosylation I: a series of 26 cases. J Med Genet. (2001) 38:14. doi: 10.1136/JMG.38.1.14, PMID: 11134235 PMC1734729

[25] Truin G Guillard M Lefeber DJ Sykut-Cegielska J Adamowicz M Hoppenreijs E. Pericardial and abdominal fluid accumulation in congenital disorder of glycosylation type Ia. Mol Genet Metab. (2008) 94:481–4. doi: 10.1016/J.YMGME.2008.05.005, PMID: 18571450

[26] Pascoal C Francisco R Mexia P Pereira BL Granjo P Coelho H. Revisiting the immunopathology of congenital disorders of glycosylation: an updated review. Front Immunol. (2024) 15:1350101. doi: 10.3389/fimmu.2024.1350101, PMID: 38550576 PMC10972870

[27] Gallego D Serrano M Cordoba-Caballero J Gámez A Seoane P Perkins JR. Transcriptomic analysis identifies dysregulated pathways and therapeutic targets in PMM2-CDG. Biochim Biophys Acta (BBA) - Mol Basis Dis. (2024) 1870:167163. doi: 10.1016/j.bbadis.2024.167163, PMID: 38599261

[28] De La Morena-Barrio ME Hernández-Caselles T Corral J García-López R Martínez-Martínez I Pérez-Dueñas B. GPI-anchor and GPI-anchored protein expression in PMM2-CDG patients. Orphanet J Rare Dis. (2013) 8:170–0. doi: 10.1186/1750-1172-8-170, PMID: 24139637 PMC4016514

[29] Bouwmeester T Bauch A Ruffner H Angrand P-O Bergamini G Croughton K. A physical and functional map of the human TNF-α/NF-κB signal transduction pathway. Nat Cell Biol. (2004) 6:97–105. doi: 10.1038/ncb1086, PMID: 14743216

[30] Gough P Myles IA. Tumor necrosis factor receptors: pleiotropic signaling complexes and their differential effects. Front Immunol. (2020) 11:585880/BIBTEX. doi: 10.3389/FIMMU.2020.585880/BIBTEX, PMID: 33324405 PMC7723893

[31] Brenner D Blaser H Mak TW. Regulation of tumour necrosis factor signalling: live or let die. Nat Rev Immunol. (2015) 15:362–74. doi: 10.1038/nri3834, PMID: 26008591

[32] MacEwan DJ. TNF ligands and receptors – a matter of life and death. Br J Pharmacol. (2002) 135:855. doi: 10.1038/SJ.BJP.0704549, PMID: 11861313 PMC1573213

[33] TNFRSF1A - Tumor necrosis factor receptor superfamily member 1A - Homo sapiens (Human). UniProtKB Available online at: https://www.uniprot.org/uniprotkb/P19438/entry. (Accessed June 05, 2025).

[34] Han L Zhang D Tao T Sun X Liu X Zhu G. The role of N-Glycan modification of TNFR1 in inflammatory microglia activation. Glycoconj J. (2015) 32:685–93. doi: 10.1007/s10719-015-9619-1, PMID: 26452604

[35] Oliveira T Ferraz R Azevedo L Quelhas D Carneiro J Jaeken J. A comprehensive update of genotype–phenotype correlations in PMM2-CDG: insights from molecular and structural analyses. Orphanet J Rare Dis. (2025) 20:207. doi: 10.1186/s13023-025-03669-5, PMID: 40307862 PMC12042452

[36] Zhang L Li W-H. Mammalian housekeeping genes evolve more slowly than tissue-specific genes. Mol Biol Evol. (2004) 21:236–9. doi: 10.1093/molbev/msh010, PMID: 14595094

[37] Palmigiano A Messina A Bua RO Barone R Sturiale L Zappia M. CSF N-glycomics using MALDI MS techniques in alzheimer’s disease. (2018), 75–91. doi: 10.1007/978-1-4939-7704-8_5, PMID: 29512066

[38] Ceroni A Maass K Geyer H Geyer R Dell A Haslam SM. GlycoWorkbench: A tool for the computer-assisted annotation of mass spectra of glycans. J Proteome Res. (2008) 7:1650–9. doi: 10.1021/pr7008252, PMID: 18311910

[39] Babraham Bioinformatics - FastQC A Quality Control tool for High Throughput Sequence Data . Available online at: https://www.bioinformatics.babraham.ac.uk/projects/fastqc/ (Accessed February 2, 2023).

[40] DOE Joint Genome Institute. . BBDuk Guide. Available online at: https://jgi.doe.gov/data-and-tools/software-tools/bbtools/bb-tools-user-guide/bbduk-guide/ (Accessed February 2, 2023).

[41] Dobin A Davis CA Schlesinger F Drenkow J Zaleski C Jha S. STAR: ultrafast universal RNA-seq aligner. Bioinformatics. (2013) 29:15–21. doi: 10.1093/BIOINFORMATICS/BTS635, PMID: 23104886 PMC3530905

[42] Anders S Pyl PT Huber W. HTSeq—a Python framework to work with high-throughput sequencing data. Bioinformatics. (2015) 31:166–9. doi: 10.1093/bioinformatics/btu638, PMID: 25260700 PMC4287950

[43] Ramilowski JA Goldberg T Harshbarger J Kloppmann E Lizio M Satagopam VP. A draft network of ligand–receptor-mediated multicellular signalling in human. Nat Commun. (2015) 6:7866. doi: 10.1038/ncomms8866, PMID: 26198319 PMC4525178

[44] Velculescu VE Madden SL Zhang L Lash AE Yu J Rago C. Analysis of human transcriptomes. Nat Genet. (1999) 23:387–8. doi: 10.1038/70487, PMID: 10581018

[45] R Core Team. R: A language and environment for statistical computing (2022). Available online at: https://www.r-project.org/. (Accessed June 05, 2025).

[46] Chen J Bardes EE Aronow BJ Jegga AG. ToppGene Suite for gene list enrichment analysis and candidate gene prioritization. Nucleic Acids Res. (2009) 37:W305–11. doi: 10.1093/nar/gkp427, PMID: 19465376 PMC2703978

[47] Jassal B Matthews L Viteri G Gong C Lorente P Fabregat A. The reactome pathway knowledgebase. Nucleic Acids Res. (2019). doi: 10.1093/nar/gkz1031, PMID: 31691815 PMC7145712

[48] GitHub. PGranjo/immunological-involvement-in-PMM2-CDG . Available online at: https://github.com/PGranjo/Immunological-Involvement-in-PMM2-CDG. (Accessed June 05, 2025).

[49] Matthijs G Schollen E Van Schaftingen E Cassiman JJ Jaeken J. Lack of homozygotes for the most frequent disease allele in carbohydrate-deficient glycoprotein syndrome type 1A. Am J Hum Genet. (1998) 62:542–50. doi: 10.1086/301763, PMID: 9497260 PMC1376957

[50] de Haas P Hendriks WJAJ Lefeber DJ Cambi A. Biological and technical challenges in unraveling the role of N-glycans in immune receptor regulation. Front Chem. (2020) 8:55. doi: 10.3389/fchem.2020.00055, PMID: 32117881 PMC7013033

[51] Wolfert MA Boons G-J. Adaptive immune activation: glycosylation does matter. Nat Chem Biol. (2013) 9:776–84. doi: 10.1038/nchembio.1403, PMID: 24231619 PMC3966069

[52] Liu J Xu X Zhong H Yu M Abuduaini N Zhang S. Glycosylation and its role in immune checkpoint proteins: from molecular mechanisms to clinical implications. Biomedicines. (2024) 12:1446. doi: 10.3390/biomedicines12071446, PMID: 39062019 PMC11274725

[53] Li X Pérez L Pan Z Fan H. The transmembrane domain of TACE regulates protein ectodomain shedding. Cell Res. (2007) 17:985–98. doi: 10.1038/cr.2007.98, PMID: 18040288

[54] Chavaroche A Cudic M Giulianotti M Houghten RA Fields GB Minond D. Glycosylation of a disintegrin and metalloprotease 17 affects its activity and inhibition. Anal Biochem. (2014) 449:68–75. doi: 10.1016/j.ab.2013.12.018, PMID: 24361716 PMC4334441

[55] Xanthoulea S Pasparakis M Kousteni S Brakebusch C Wallach D Bauer J. Tumor necrosis factor (TNF) receptor shedding controls thresholds of innate immune activation that balance opposing TNF functions in infectious and inflammatory diseases. J Exp Med. (2004) 200:367–76. doi: 10.1084/jem.20040435, PMID: 15289505 PMC2211976

[56] Parsons PE Matthay MA Ware LB Eisner MD. Elevated plasma levels of soluble TNF receptors are associated with morbidity and mortality in patients with acute lung injury. Am J Physiology-Lung Cell Mol Physiol. (2005) 288:L426–31. doi: 10.1152/ajplung.00302.2004, PMID: 15516488

[57] Chemaly M Gibson M Watterson S Bjourson T McGilligan V Peace A. P6229TACE gene expression and soluble receptors TNFRI and TNFRII levels identifies very high risk cardiovascular patients. Eur Heart J. (2017) 38. doi: 10.1093/eurheartj/ehx493.P6229

[58] Horiuchi K. A brief history of tumor necrosis factor α – converting enzyme: an overview of ectodomain shedding. Keio J Med. (2013) 62:29–36. doi: 10.2302/kjm.2012-0003-RE, PMID: 23563789

[59] Kirkegaard T Pedersen G Saermark T Brynskov J. Tumour necrosis factor- α converting enzyme (TACE) activity in human colonic epithelial cells. Clin Exp Immunol. (2003) 135:146–53. doi: 10.1111/j.1365-2249.2004.02348.x, PMID: 14678276 PMC1808921

[60] Chanthaphavong RS Loughran PA Lee TYS Scott MJ Billiar TR. A role for cGMP in inducible nitric-oxide synthase (iNOS)-induced tumor necrosis factor (TNF) α-converting enzyme (TACE/ADAM17) activation, translocation, and TNF receptor 1 (TNFR1) shedding in hepatocytes. J Biol Chem. (2012) 287:35887–98. doi: 10.1074/jbc.M112.365171, PMID: 22898814 PMC3476257

[61] Xue Y Zeng X Tu W-J Zhao J. Tumor necrosis factor-α: the next marker of stroke. Dis Markers. (2022) 2022:1–8. doi: 10.1155/2022/2395269, PMID: 35265224 PMC8898850

[62] Pan W Kastin AJ. Tumor necrosis factor and stroke: Role of the blood–brain barrier. Prog Neurobiol. (2007) 83:363–74. doi: 10.1016/j.pneurobio.2007.07.008, PMID: 17913328 PMC2190541

[63] Izquierdo-Serra M Martínez-Monseny A López L Carrillo-García J Edo A Ortigoza-Escobar J. Stroke-Like Episodes and Cerebellar Syndrome in Phosphomannomutase Deficiency (PMM2-CDG): Evidence for Hypoglycosylation-Driven Channelopathy. Int J Mol Sci (2018) 19:619. doi: 10.3390/ijms19020619, PMID: 29470411 PMC5855841

[64] Radovani B Gudelj I. N-glycosylation and inflammation; the not-so-sweet relation. Front Immunol. (2022) 13:893365. doi: 10.3389/FIMMU.2022.893365, PMID: 35833138 PMC9272703

[65] Dewald JH Colomb F Bobowski-Gerard M Groux-Degroote S Delannoy P. Role of cytokine-induced glycosylation changes in regulating cell interactions and cell signaling in inflammatory diseases and cancer. Cells. (2016) 5. doi: 10.3390/CELLS5040043, PMID: 27916834 PMC5187527

[66] McCarthy C Saldova R Wormald MR Rudd PM McElvaney NG Reeves EP. The role and importance of glycosylation of acute phase proteins with focus on alpha-1 antitrypsin in acute and chronic inflammatory conditions. J Proteome Res. (2014) 13:3131–43. doi: 10.1021/PR500146Y, PMID: 24892502

[67] Thiesler CT Cajic S Hoffmann D Thiel C Van Diepen L Hennig R. Glycomic characterization of induced pluripotent stem cells derived from a patient suffering from phosphomannomutase 2 congenital disorder of glycosylation (PMM2-CDG). Mol Cell Proteomics. (2016) 15:1435–52. doi: 10.1074/MCP.M115.054122, PMID: 26785728 PMC4824866

[68] Sharma V Ichikawa M Freeze HH. Mannose metabolism: more than meets the eye. Biochem Biophys Res Commun. (2014) 453:220. doi: 10.1016/J.BBRC.2014.06.021, PMID: 24931670 PMC4252654

[69] Yoldas Celik M Yazici H Erdem F Yuksel Yanbolu A Aykut A Durmaz A. Unique clinical presentations and follow-up outcomes from experience with congenital disorders of glycosylation: PMM2-PGM1-DPAGT1-MPI-POMT2-B3GALNT2-DPM1-SRD5A3-CDG. J Pediatr Endocrinol Metab. (2023) 36:530–8. doi: 10.1515/JPEM-2022-0641, PMID: 37042760

[70] Lavoie H Gagnon J Therrien M. ERK signalling: a master regulator of cell behaviour, life and fate. Nat Rev Mol Cell Biol. (2020) 21:607–32. doi: 10.1038/S41580-020-0255-7, PMID: 32576977

[71] Dasi S Choi J Lambertz I Kelliher MA Eliopoulos AG Du K. Tpl2/cot signals activate ERK, JNK, and NF-kappaB in a cell-type and stimulus-specific manner. J Biol Chem. (2005) 280:23748–57. doi: 10.1074/JBC.M412837200, PMID: 15833743

[72] Rose-John S Winthrop K Calabrese L. The role of IL-6 in host defence against infections: immunobiology and clinical implications. Nat Rev Rheumatol. (2017) 13:399–409. doi: 10.1038/NRRHEUM.2017.83, PMID: 28615731

[73] Levy JA. The unexpected pleiotropic activities of RANTES. J Immunol. (2009) 182:3945–6. doi: 10.4049/JIMMUNOL.0990015, PMID: 19299688

[74] He P Srikrishna G Freeze HH. N-glycosylation deficiency reduces ICAM-1 induction and impairs inflammatory response. Glycobiology. (2014) 24:392–8. doi: 10.1093/GLYCOB/CWU006, PMID: 24474243 PMC3954120

[75] Davis RJ. Signal transduction by the JNK group of MAP kinases. Cell. (2000) 103:239–52. doi: 10.1016/S0092-8674(00)00116-1, PMID: 11057897

[76] Kim C Sano Y Todorova K Carlson BA Arpa L Celada A. p38α MAP kinase serves cell type-specific inflammatory functions in skin injury and coordinates pro- and anti-inflammatory gene expression. Nat Immunol. (2008) 9:1019. doi: 10.1038/NI.1640, PMID: 18677317 PMC2587092

[77] Gilley R March HN Cook SJ. ERK1/2, but not ERK5, is necessary and sufficient for phosphorylation and activation of c-Fos. Cell Signal. (2009) 21:969–77. doi: 10.1016/J.CELLSIG.2009.02.006, PMID: 19249353

[78] Plotnikov A Zehorai E Procaccia S Seger R. The MAPK cascades: Signaling components, nuclear roles and mechanisms of nuclear translocation. Biochim Biophys Acta (BBA) - Mol Cell Res. (2011) 1813:1619–33. doi: 10.1016/J.BBAMCR.2010.12.012, PMID: 21167873

[79] Angel P Szabowski A Schorpp-Kistner M. Function and regulation of AP-1 subunits in skin physiology and pathology. Oncogene. (2001) 20:2413–23. doi: 10.1038/SJ.ONC.1204380, PMID: 11402337

[80] Saha RN Jana M Pahan K. MAPK p38 Regulates Transcriptional Activity of NF-κB in Primary Human Astrocytes via Acetylation of p65. J Immunol (2007) 179:7101–9. doi: 10.4049/jimmunol.179.10.7101, PMID: 17982102 PMC2701546

[81] Pascoal C Granjo P Mexia P Gallego D Adubeiro Lourenço R Sharma S. Unraveling the biological potential of skin fibroblast: responses to TNF-α, highlighting intracellular signaling pathways and secretome. Immunol Lett. (2025) 276:107057. doi: 10.1016/j.imlet.2025.107057, PMID: 40619104

[82] Edmondson AC Honzík T Lam C Õunap K McWilliams P Morava E. Incidence and prevalence of phosphomannomutase 2-congenital disorder of glycosylation: Past, present, and future. Mol Genet Metab. (2025) 146:109188. doi: 10.1016/j.ymgme.2025.109188, PMID: 40737785

[83] Committee for Medicinal Products for Human Use (CHMP). Guideline on clinical trials in small populations. London (2006). Available online at: https://www.ema.europa.eu/en/clinical-trials-small-populations-scientific-guideline.

