# Supplementary material for: Immunopathology in PMM2-CDG: Defective glycosylation impact in the TNFα -TNFR1 signalling pathway
Source: Front Immunol. 2025 Sep 18;16:1655354. doi: 10.3389/fimmu.2025.1655354 (PMC12488661; doi:10.3389/fimmu.2025.1655354)
Supplement: Supplementary file 7 [file DataSheet1.pdf]

Supplementary Material

This PDF file includes:

Figures S1 to S6

Other supporting materials for this manuscript include the following:

Tables S1 to S6

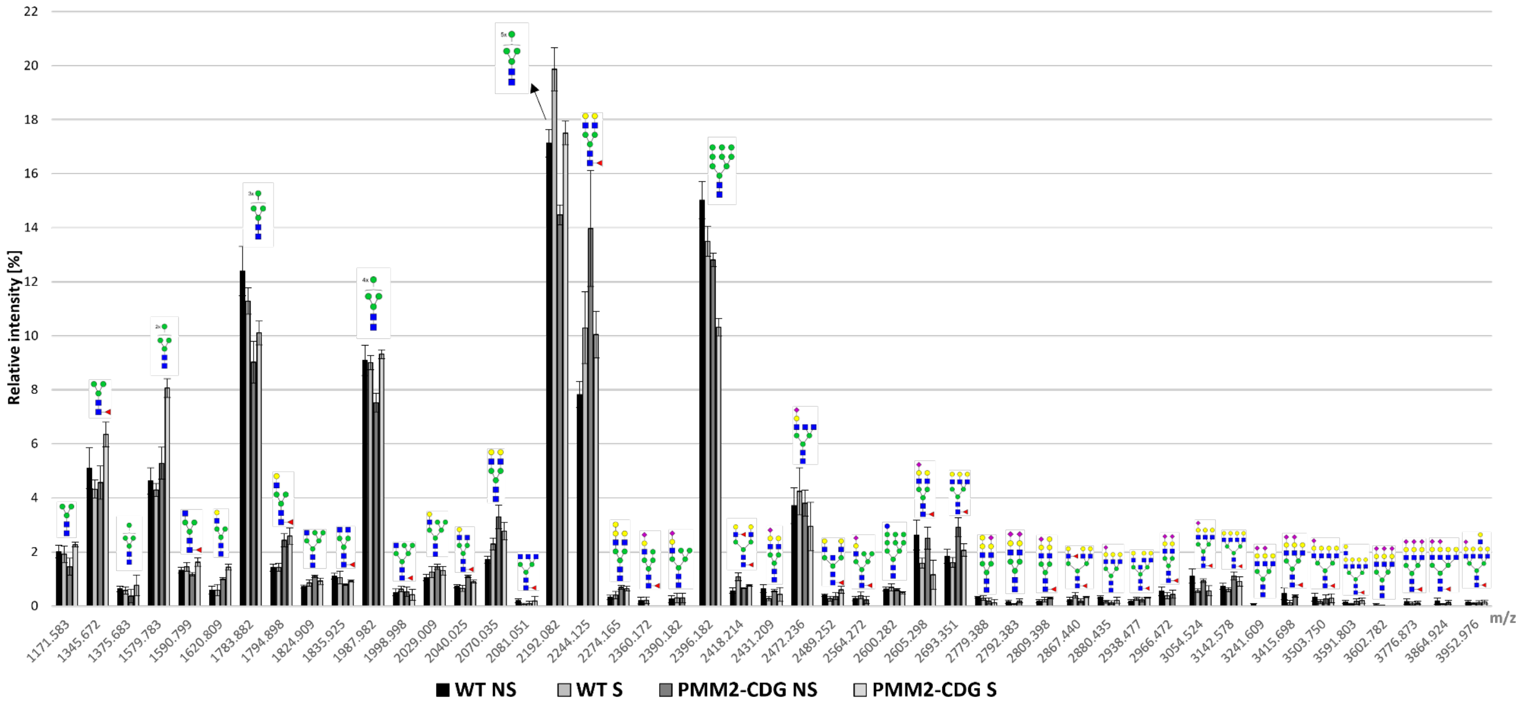

Figure S1. Relative intensities of 47 individual N-glycan structures identified in lysed fibroblasts by MALDI-TOF mass spectrometry. Data were acquired in reflectron positive ion mode after glycan release, isolation and permethylation. Green circle – mannose, yellow circle – galactose, blue square – N-acetylglucosamine, red triangle – fucose, purple diamond – sialic acid.

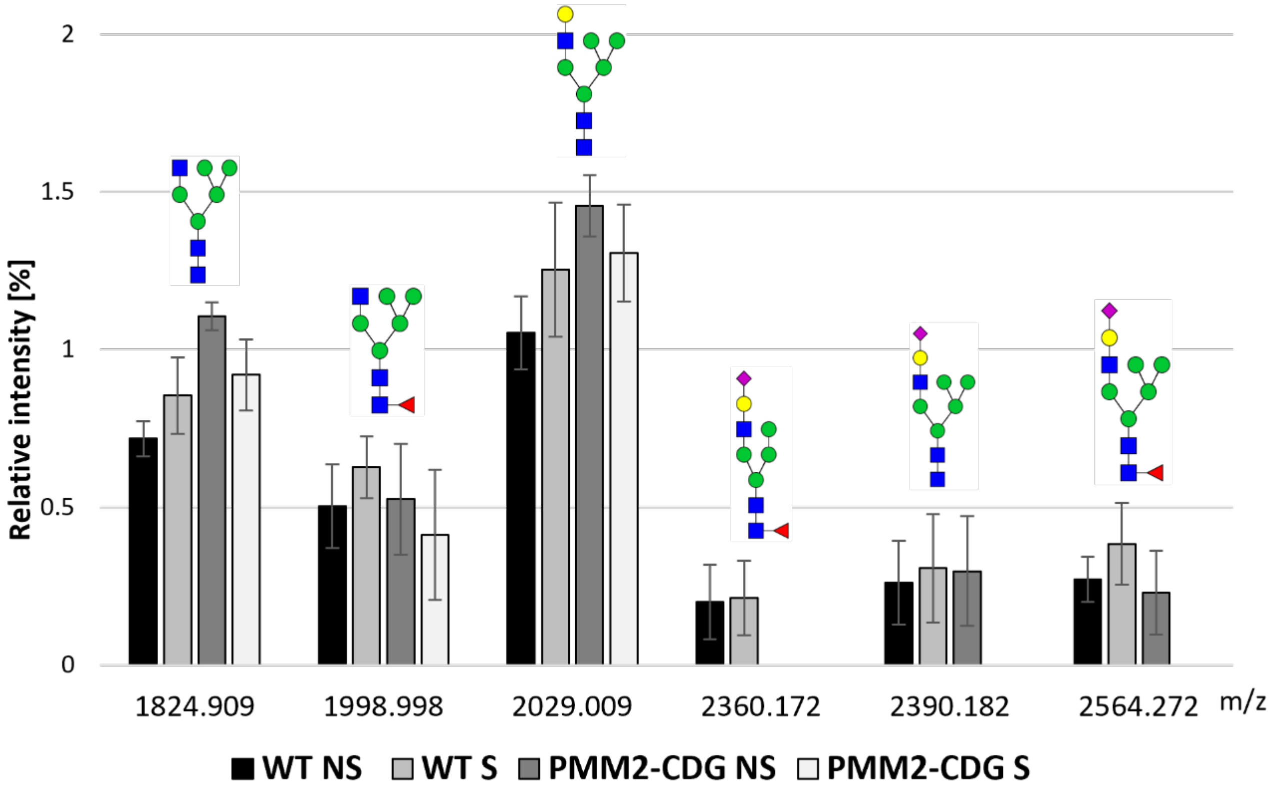

Figure S2. Relative intensities of hybrid N-glycan structures identified in lysed fibroblasts by MALDI-TOF mass spectrometry. Green circle – mannose, yellow circle – galactose, blue square – N-acetylglucosamine, red triangle – fucose, purple diamond – sialic acid.

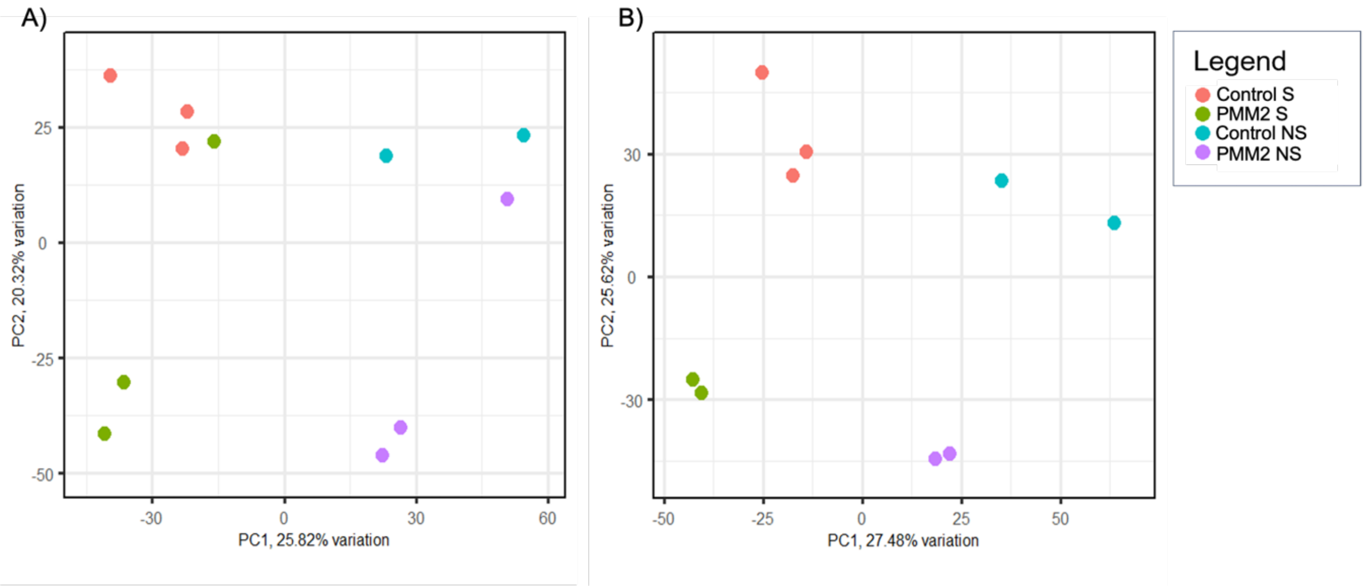

Figure S3. Principal component analysis biplots of control (healthy individuals) and PMM2-CDG samples before and upon TNF-α stimulus of (A) all control and PMM2-CDG stimulated and non-stimulated samples and (B) of control and PMM2-CDG samples bearing the heterozygous genetic variant p.R141H (GM27226 and GM27386). Legend: PC – principal component; S – Stimulated; NS – Non-stimulated.

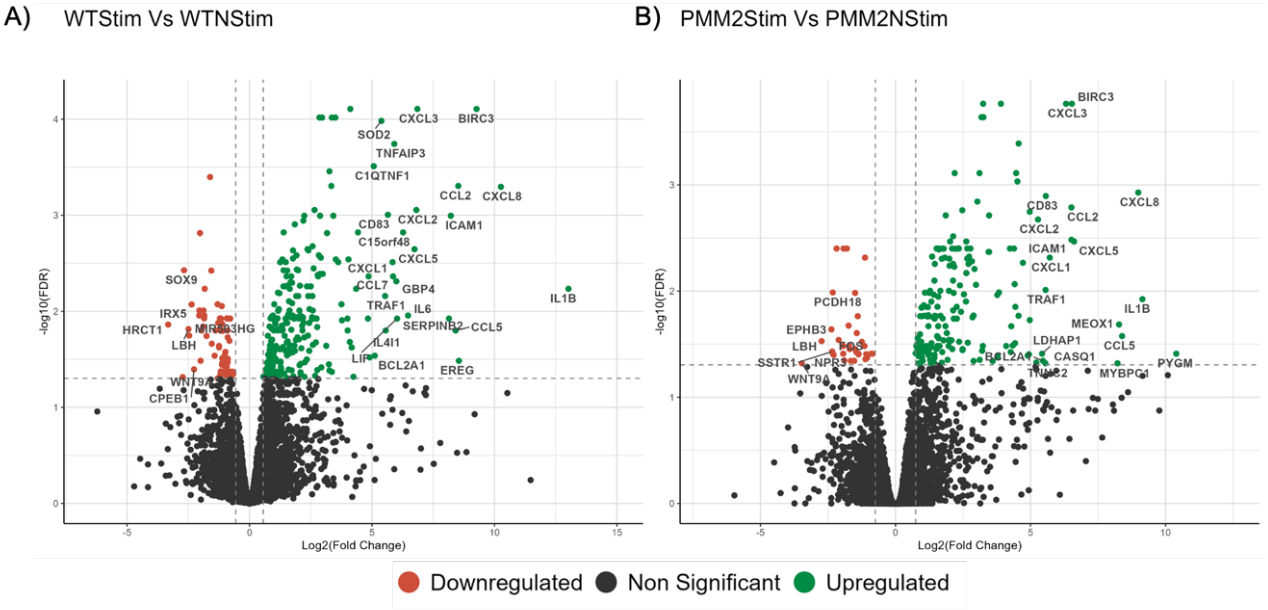

Figure S4. Volcano plot of the differential expressed genes (DEGs) of control fibroblasts (left) and PMM2-CDG (right) fibroblasts upon TNF-α stimulus. The cut of criteria to select the DEGs was an adj. P-value < 0.05. Red dots represent all the DEGs (P-value < 0.05 and log2FC > 0.56); black dots represent all the genes with a log2FC > 0.56 but that are not significantly different before and after stimulation (adj. P-value > 0.05); lastly, grey dots comprise all non-differentially expressed genes with a log2FC < 0.56. The 0.56 of Log2FC was selected due to being the lowest log2FC in both groups.

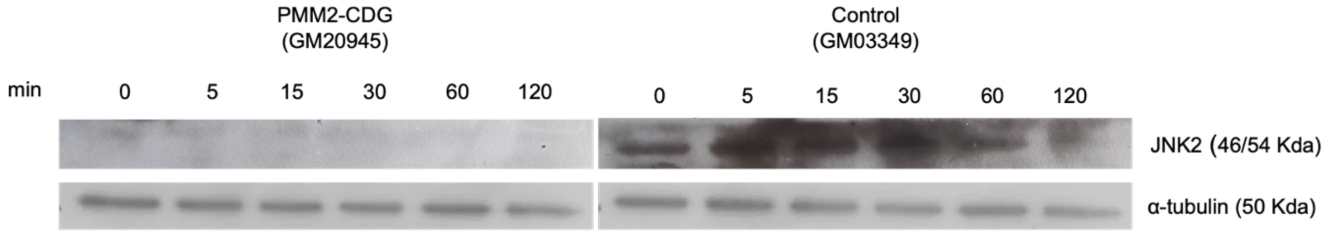

Figure S5. Western blot analysis of the JNK2 protein in PMM2-CDG and control skin fibroblasts. Fibroblasts were stimulated with 10 ng/ml of TNF-α for increasing periods of time. Immunoblotting was performed using anti-JNK2 (Cell Signaling) at 1:1000. Mouse monoclonal anti-α-tubulin staining was performed as loading control.
